# Structural and Synthetic Aspects of Small Ring Oxa- and Aza-Heterocyclic Ring Systems as Antiviral Activities

**DOI:** 10.3390/v15091826

**Published:** 2023-08-28

**Authors:** Sibasish Manna, Koushik Das, Sougata Santra, Emily V. Nosova, Grigory V. Zyryanov, Sandipan Halder

**Affiliations:** 1 Department of Chemistry, Visvesvaraya National Institute of Technology, Nagpur 440010, India; 2 Department of Organic and Biomolecular Chemistry, Chemical Engineering Institute, Ural Federal University, 19 Mira Street, 620002 Yekaterinburg, Russia; sougatasantra85@gmail.com (S.S.); emilia.nosova@yandex.ru (E.V.N.); g.v.zyrianov@urfu.ru (G.V.Z.); 3 I. Ya. Postovskiy Institute of Organic Synthesis, Ural Division of the Russian Academy of Sciences, 22 S. Kovalevskoy Street, 620219 Yekaterinburg, Russia

**Keywords:** antiviral agents, heterocycles, natural products, *Structure Activity Relationship* (SAR), synthetic methods

## Abstract

Antiviral properties of different oxa- and aza-heterocycles are identified and properly correlated with their structural features and discussed in this review article. The primary objective is to explore the activity of such ring systems as antiviral agents, as well as their synthetic routes and biological significance. Eventually, the *structure–activity relationship* (SAR) of the heterocyclic compounds, along with their salient characteristics are exhibited to build a suitable platform for medicinal chemists and biotechnologists. The synergistic conclusions are extremely important for the introduction of a newer tool for the future drug discovery program.

## 1. Introduction

**Figure 1:**
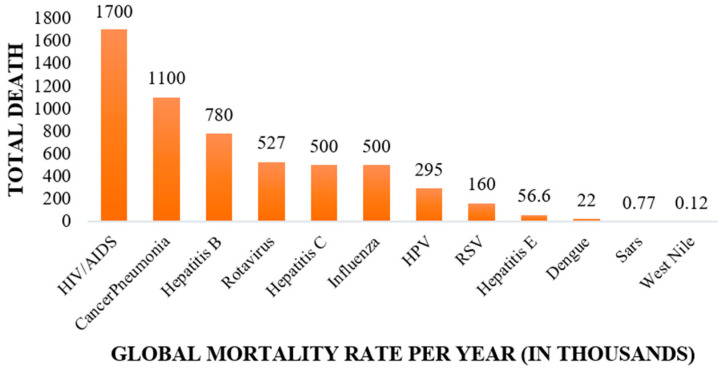
Global mortality rate per year due to different viral infections [13].

The terrible impact of viral diseases has become a severe concern for the whole animal kingdom, including human beings, during the last few decades [1,2,3]. Several categories of viruses, given their diverse behavior against biological systems, are the major reasons for the incidence of chronic health issues. It is indeed extremely important to address the epidemic nature and the corresponding mortality rate of such diseases in comparison to other fatal infections [4,5]. According to recent reports, the highest number of deaths have been caused by cardiovascular diseases throughout the globe [6], but still, viral infections also have been responsible for millions of human casualties every year (Figure 1). Human immunodeficiency virus (HIV) is considered to be very fatal in nature [7], but it is also very important to note the pervasive nature of other viruses with respect to geographic and economic diversity [8]. As, for example, Rabies disease (originated from domestic dogs) is 100% fatal to humans, but not a pandemic [9]. Disease due to the Ebola virus is a global pandemic with very high average fatality rate (~55%), but it varies depending upon the region [10,11,12]. 

In the year of 1918, there was an outbreak of the H1N1 Influenza virus, which is definitely considered to be the world’s biggest pandemic, known as the Spanish flu [14,15]. It was exceptionally severe; 50 million people (~2% of the world’s population) become infected during 1918–1920 [16,17]. In recent times, the entire world has gone through a catastrophic situation due to the rapid spread of a newly emerged COVID-19, or severe acute respiratory syndrome CoV-2 (SARS-CoV-2) virus [18]. The existence of this highly contagious virus was first reported in December 2019 in Wuhan, China. The World Health Organization (WHO) began immediate special surveillance on this particular issue and effectively framed several protocols throughout the globe. But to the present day, there are very limited remedies available to fight against this COVID-19 [19,20,21,22,23]. The vaccines listed and recommended by WHO are the only solutions to combat these deadly infections [24]. After facing such situations, it is undoubtedly needed to develop libraries of newer drug candidates corresponding to each and every virus, in order to control their fatal outcomes.

In drug discovery research, the role of heterocyclic moieties is extremely significant in order to make stable interactions with the targeted proteins [25,26]. Such ring systems offer suitable coordination with the specific proteins by tailoring the bulk size and pertinent electron density to modulate the efficacy of the drug compounds in the biological environments [27,28,29,30,31,32]. For these reasons, the synthetic and biological studies of structurally designed heterocyclic moieties are really a crucial measure for the development of future generation drug candidates [33,34,35,36,37]. In this review article, our main focus will be on the structural features, along with the synthetic routes, of oxygen- and nitrogen-containing ring systems (saturated/unsaturated derivatives) present in several natural products, marketed drugs and synthetic analogues having prominent antiviral activities.

## 2. Overview of the Viral Diseases

Based on its genomic aspects, a virus could be classified as a DNA virus [38] (DNA as a genetic material), which replicates with the help of DNA polymerase (e.g., HSV and HCMV). Secondly, it could be categorized as an RNA virus (RNA as a genetic material), which replicates in the presence of RNA polymerase (e.g., HCV, HBV, RSV and Ebola virus) [39]. Thirdly, it could be categorized as a reverse-transcribing virus [40]; for these viruses, the genome is RNA, but by using a reverse transcriptase enzyme, it is able to form DNA (e.g., HIV) [41].

### 2.1. Human Immunodeficiency Virus (HIV)

This retrovirus was discovered in the year 1983 [42] and is mainly transmitted through bodily fluids or by bodily contacts of HIV-positive patients. According to the WHO global health survey, there are 38.0 million people living with HIV, and 690,000 people had died by the year 2021 [43]. There is no specific vaccine or medicine available to cure AIDS, but there are some natural and synthetic drug candidates available [44,45]. Dolutegravir, sold under the brand name of Tivicay, was the bestselling anti-HIV drug in 2018 [46]. Some other synthetic drugs like zalcitabine [47], zidovudine [48] and emtricitabine [49] terminate the viral DNA chain by inhibiting the reverse transcriptase.

### 2.2. Hepatitis C Virus (HCV)

This is a blood-borne virus, discovered in 1989, which generally is transmitted in a similar way to HIV [50]. According to the WHO global health survey, hepatitis C generates chronic diseases like liver cirrhosis and jaundice. Till now there is no specific vaccine or medicine available for the treatment of hepatitis C. There are some marketed drugs available for the general treatment of HCV. 

Mavyret, which is the composition of glecaprevir and pibrentasvir, was the best-selling anti-HCV drug in 2018 [51]. Asunaprevir, boceprevir and grazoprevir inhibit the proteolytic activity of HCV NS3/4A protease and show promising anti-HCV properties [52,53,54].

### 2.3. Hepatitis B Virus (HBV)

The existence of this virus was confirmed in the year of 1963; it generally is transmitted through sexual contact, blood transfusion or by bodily fluids [55]. According to the WHO global health survey, HBV causes approximately 780,000 deaths every year. The vaccine that corresponds to HBV is available in the market as a hepatitis B surface antigen (HBsAg) [56]; along with that, there are several other drugs that are also available in the market for the treatment of hepatitis B, with the names entecavir, telbivudine and lamivudine [57,58,59].

### 2.4. Respiratory Syncytial Virus (RSV)

The World Health Organization (WHO) has reported that RSV causes a significant number of casualties each year, ranging from 66,000 to 199,000 [60]. Moreover, in the year 2005, it was estimated that RSV had infected around 33.8 million children [61,62]. This virus is transmitted through the respiratory systems, by droplets, or from contaminated substances [63].

### 2.5. Human Cytomegalovirus (HCMV)

HCMV, a member of the herpes virus family, is a prevalent virus that often presents with mild or no symptoms in healthy individuals [64]. Moreover, individuals with weakened immune systems, including newborns, pregnant women and immune compromised individuals can experience severe complications. This infectious virus generally spreads with the help of bodily fluids; it causes serious organ damage, including gastrointestinal problems and colitis [65]. The management of HCMV infection involves the use of antiviral medications which effectively control the virus and alleviate symptoms [66].

### 2.6. Herpes Simplex Virus (HSV)

Herpes simplex virus (HSV) is a highly contagious virus that causes recurrent infections. The virus is transmitted through direct contact with an infected person’s skin, mucous membranes, or bodily fluids [67]. Although there is no cure for HSV, antiviral medications can help to manage symptoms and reduce the frequency and severity of outbreaks. According to the WHO global health survey (2016), over half a billion people worldwide are estimated to have genital herpes caused by herpes simplex virus type 1 (HSV-1) or type 2 (HSV-2) [68].

### 2.7. Ebola Virus (EBOV)

The existence of the Ebola virus was first understood in the year of 1976 [69]. The primary mode of transmission for the Ebola virus is through direct contact with infected blood, bodily fluids, or tissues [70]. In the year 2018, WHO documented approximately 11,500 deaths globally attributed to Ebola. However, it is important to note that these figures are not fixed and can fluctuate over time and across different regions. The impact of Ebola outbreaks can vary depending on factors such as healthcare infrastructure, access to resources, and public health interventions. Efforts are continuously underway to improve surveillance and response capabilities and institute preventive measures to minimize the spread of the virus and reduce the number of Ebola-related fatalities.

### 2.8. Severe Acute Respiratory Syndrome CoV-2 (SARS-CoV-2) Virus

Coronaviruses are positive single-stranded RNA viruses that have an enveloped structure and can infect humans and various animals [71]. In 1965, Tyrrell and Bynoe made a significant contribution to the history of human coronaviruses [72]. They discovered that a virus named B814, obtained from the respiratory tract of an adult with a common cold, could be successfully propagated in human embryonic tracheal organ cultures. This discovery paved the way for further research on coronaviruses and their potential impact on human health [73]. These viruses have a spherical shape, with surface projections resembling the solar corona, hence their name “coronaviruses” (derived from the Latin word “corona”, meaning “crown”). There are four subfamilies of coronaviruses, including alpha, beta, gamma and delta. The alpha and beta variants likely originate from mammals, predominantly bats, while gamma and delta are associated with pigs and birds. Coronaviruses have genome sizes ranging from 26 to 32 kb. Among the seven subtypes of coronaviruses that can infect humans, beta-coronaviruses can lead to severe illness and fatalities, whereas alpha-coronaviruses typically result in mild or asymptomatic infections [74].

### 2.9. The Human Papillomavirus (HPV)

The human papillomavirus is a common virus that can infect the body’s skin and mucous membranes. It is usually transferred through sexual contact or skin-to-skin contact [75]. According to the WHO report, the consequence of this virus is very much prominent in patients with cervical cancer; the majority of the deaths of such women patients are due to a HPV infection [76].

### 2.10. Rabies Virus

Rabies virus is a deadly virus that affects the central nervous system of mammals, including humans. It primarily spreads through the saliva of infected animals, usually through a bite. The virus is believed to have originated from bats, but is also found in other animals such as dogs, cats and other wild animals (e.g., fox, raccoon and skunk) [77].

### 2.11. Zika Virus

The Zika virus is a mosquito-borne virus that was first identified in the Zika Forest of Uganda in 1947 [78]. It remained relatively unknown until a major outbreak occurred in the year 2015 in Brazil, which rapidly spread throughout the Americas and the Caribbean [79].

Most people infected by the Zika virus experience mild symptoms, and the incidence of casualties is also rare, but the viral infection is found to link with birth defects in newborn babies. In most cases, it causes microcephaly, in which the infants are born with abnormally small or underdeveloped brains [80]. The exact number of deaths related to the Zika virus is difficult to determine, as many deaths may have been caused by co-infections or other complications [81].

### 2.12. The Poliovirus

The poliovirus was first isolated in 1909 by Karl Landsteiner and Erwin Popper; it is a highly contagious virus that primarily affects young children and can lead to paralysis, and even death [82]. It is believed to have originated in ancient times and has been a major public health concern worldwide since the early 20th century [83].

Polio generally is transmitted through contaminated food and water or direct contact with an infected person’s saliva [84]. As per the WHO report, the Global Polio Eradication Initiative has led to an impressive 99% reduction in polio infections. In 2020, only 140 polio cases were found worldwide [85].

### 2.13. West Nile Virus

West Nile virus (WNV) is a virus that generally enters into humans through the bite of infected mosquitoes. The virus originated in Africa, and was first identified in the West Nile district of Uganda in 1937 [86]. The virus can cause a range of symptoms, from mild flu to more severe neurological problems like meningitis and encephalitis [87].

### 2.14. The Chickenpox Virus

The virus that corresponds to chickenpox is known as varicella–zoster virus (VZV), which is a highly contagious virus that causes a characteristic itchy rash and fever. The virus mostly spreads through respiratory droplets or direct contact with the fluid from the blisters of infected individuals [88].

### 2.15. The Influenza Virus

The influenza virus is a highly infectious respiratory virus that can cause mild to severe illness, and even death. It is believed that this virus originated in birds and transmitted to humans through close contact with the infected birds or contaminated surfaces [89]. The severity of the illness varies from a mild fever to severe symptoms, or even death. Globally, this annual epidemic leads to approximately three to five million cases of severe illness each year [90].

### 2.16. Yellow Fever

Yellow fever virus is a Flavivirus which originated in Africa and is transmitted by the Aedes aegypti mosquito. The virus can cause a wide range of symptoms, from mild flu to hemorrhagic fever [91]. Yellow fever is endemic in tropical regions of Africa and South America, where it affects thousands of people every year. The majority of infections are asymptomatic, but in severe cases, the virus can cause liver damage, kidney failure and death. According to the WHO report, yellow fever still causes an estimated 200,000 cases and 30,000 deaths per year, mostly in the tropical regions where it is endemic [92].

## 3. Overview of the Antiviral Drugs

**Figure 2:**
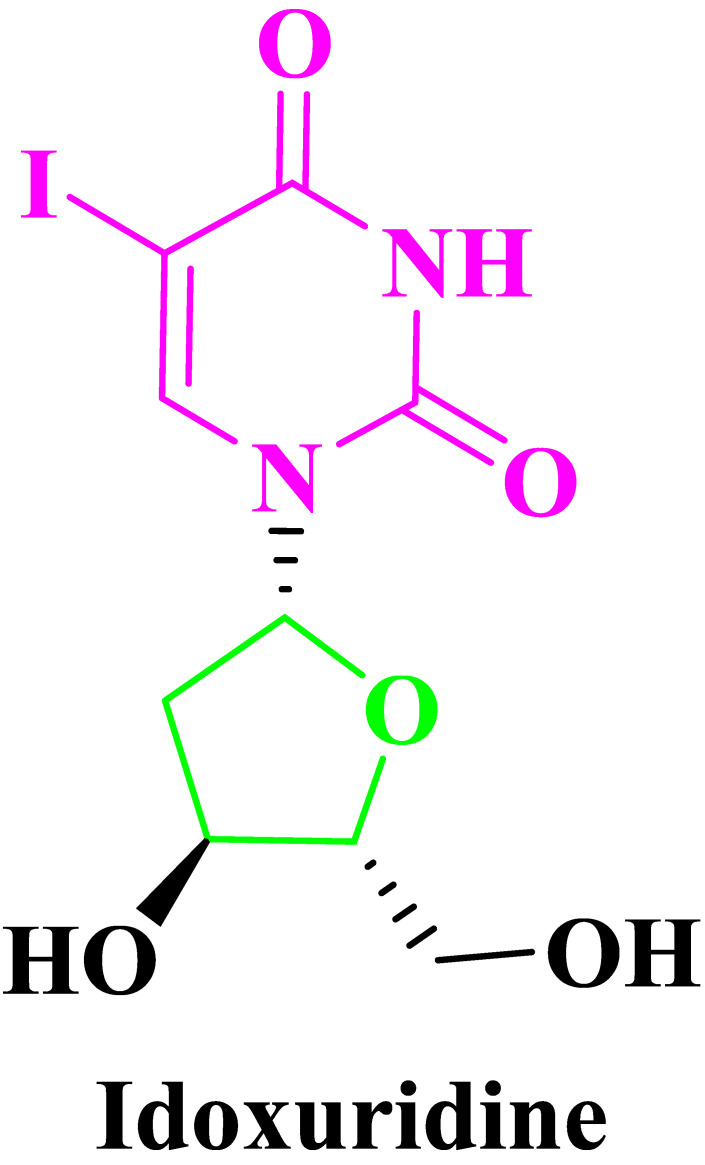
First FDA-approved antiviral drug Idoxuridine.

The period of antiviral drugs begins from the year 1959 with the introduction of *Idoxuridine* (5-iodo-2′-deoxyuridine) (Figure 2), the first antiviral drug (Figure 2), by the American pharmacologist William H. Prusoff for the treatment of HSV keratitis in humans [93,94,95]. This drug was formally approved by FDA in June of 1963; subsequently, different categories of drugs have been discovered and marketed to combat other viral infections [96]. 

### 3.1. Representative Antiviral Drug Candidates

**Figure 3:**
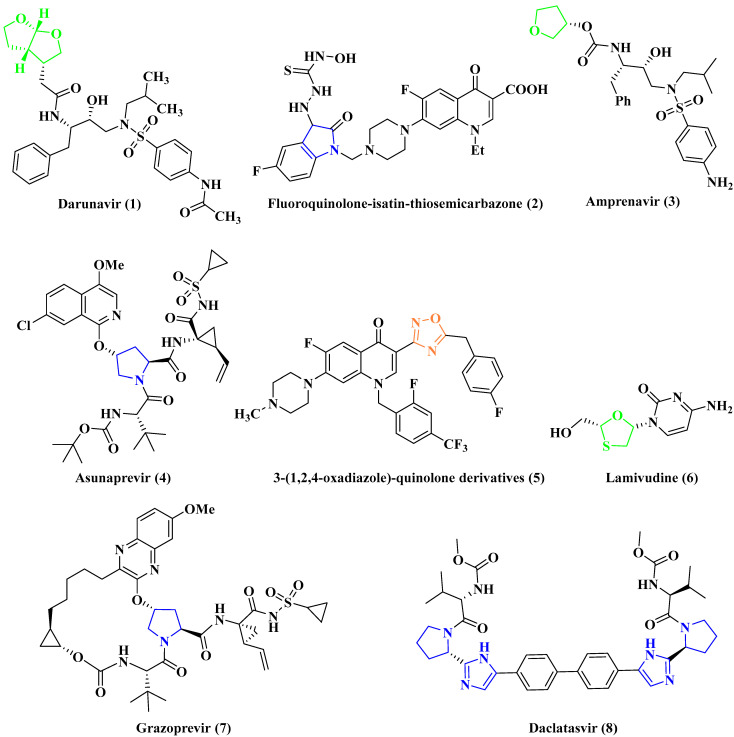

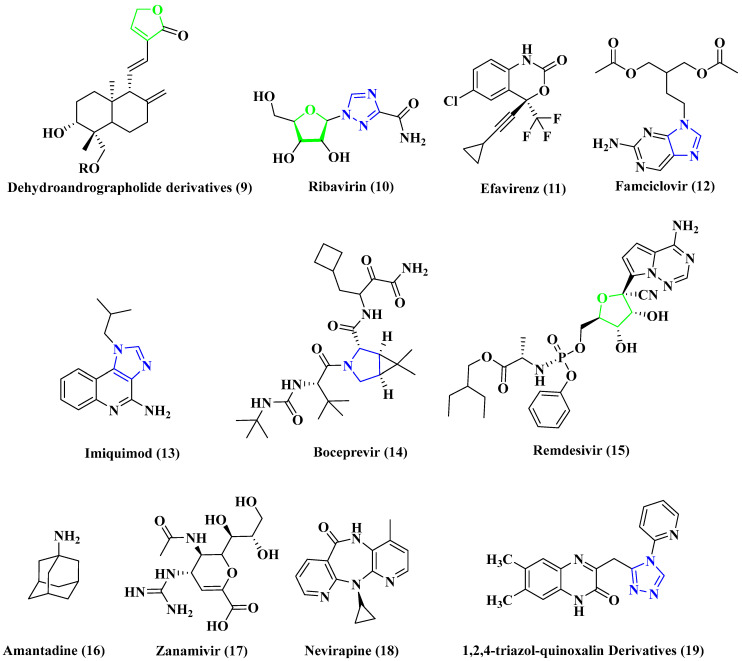
Representative examples of antiviral drug candidates.

Antiviral drugs (Figure 3) target specific enzymes and proteins involved in the viral life cycle, such as RNA-dependent DNA polymerase, RNA-dependent RNA polymerase, proteases and neuraminidases. RNA-dependent DNA polymerase inhibitors are used for retroviral infections like HIV, while RNA-dependent RNA polymerase inhibitors are efficient against RNA viral infections. Protease inhibitors are effective against viruses that require proteases, and neuraminidase inhibitors treat influenza [97].

### 3.2. Antiviral Drugs Containing Nucleoside Subunit

**Figure 4:**
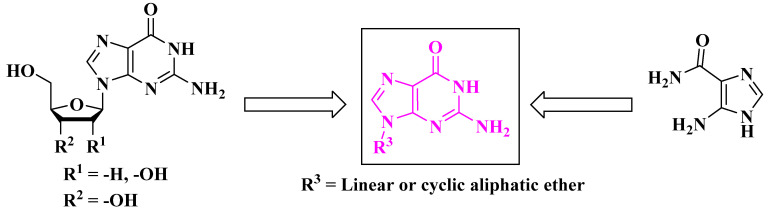

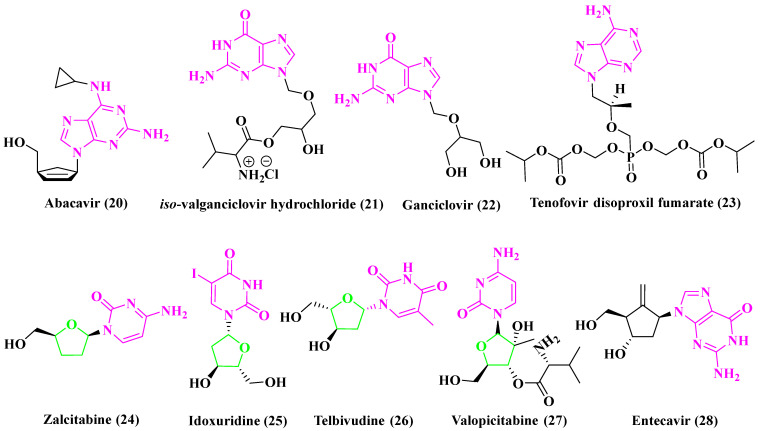
Representative nucleoside drugs.

These drugs contain nucleobase/substituted nucleobase and a sugar derivative (Figure 4) having prominent antiviral properties. Arabinosyl nucleoside analogues were isolated initially from sponges [98].

### 3.3. Examples of Natural Products with Antiviral Properties

**Figure 5:**
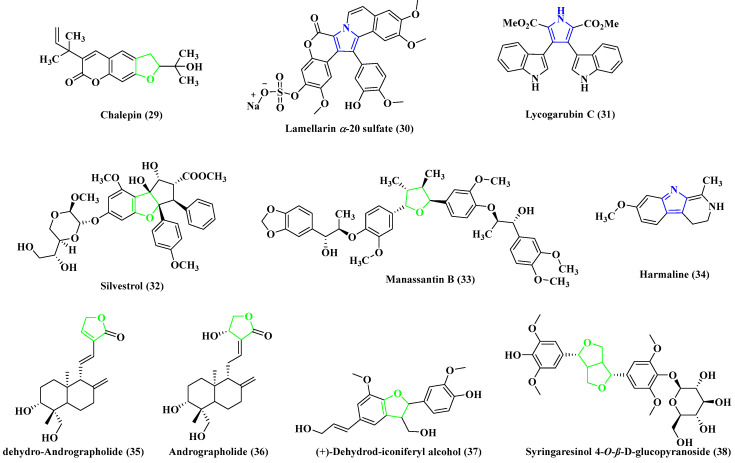
Representative natural products having antiviral activities.

Nature is a continuous source for the provision of different kinds of natural products having excellent biological activities (Figure 5). The alkaloids found in numerous plants and marine algae show important antiviral properties.

- Chalepin (**29**), from a *Ruta angustifolia* species plant, shows a good inhibitory effect against HCV [99].
- Lamellarin *α*-20 sulfate (**30**) is an alkaloid found in marine Lamellarins [100], and is responsible for inhibiting the integration of HIV-1 replication in its very early stages.
- Lycogarubins (A, B and C) are isolated from fruit bodies of Myxomycetes *Lycogala epidendnrm*, and contain two indole groups connected with dimethyl pyrrole-dicarboxylate, in which Lycogarubin C (**31**) shows activity against HSV [101].
- Silvestrol (**32**), from the bark of the *Aglaia foveolate* type of plants, contains a substituted dioxane and acts as a potent inhibitor of the Ebola virus [102].
- Manassantin B (**33**), extracted from *Saururus chinensis Baill* plants, shows inhibitory properties against the Epstein–Barr virus [103].
- Harmaline (**34**) is an indole alkaloid from *Peganum harmala*, and shows antiviral properties against HSV-1 [104].
- Dehydro-Andrographolide (**35**) and Andrographolide (**36**) are two types of natural diterpenoids that have been extracted from *Andrographis paniculata*. These compounds have demonstrated the ability to inhibit the replication of HBV DNA [105].
- (+)-Dehydrod-iconiferyl alcohol (**37**) that has been isolated from *Swertia patens* shows inhibitory activities on the secretion of HBsAg, with IC_50_ value of 1.94 mM [106].
- Syringaresinol 4″-*O*-*β*-D-glucopyranoside (**38**), which was extracted from *Swertia chirayita*, exhibited an inhibitory effect on the secretion of HBsAg, with IC_50_ values of 1.49 ± 0.033 mM [107].

## 4. Importance of Heterocyclic Ring Systems as Antiviral Agents

Nitrogen- and oxygen-containing ring systems offer significant activities as antiviral candidates [108,109,110,111]. These ring systems have either been constructed by linear synthetic steps or could be present from particular starting substrates (like sugar or aza-sugar derivatives). Several research groups have made immense efforts seeking the development of such heterocyclic subunits. Here, the heterocyclic moieties have been screened based on their profound antiviral properties and the corresponding synthetic protocols have been discussed.

## 5. Synthetic Outlines of Representative Antiviral Drug Candidates

### 5.1. Anti-HIV Agent

#### 5.1.1. Anti-HIV Agent Darunavir

**Figure 6:**
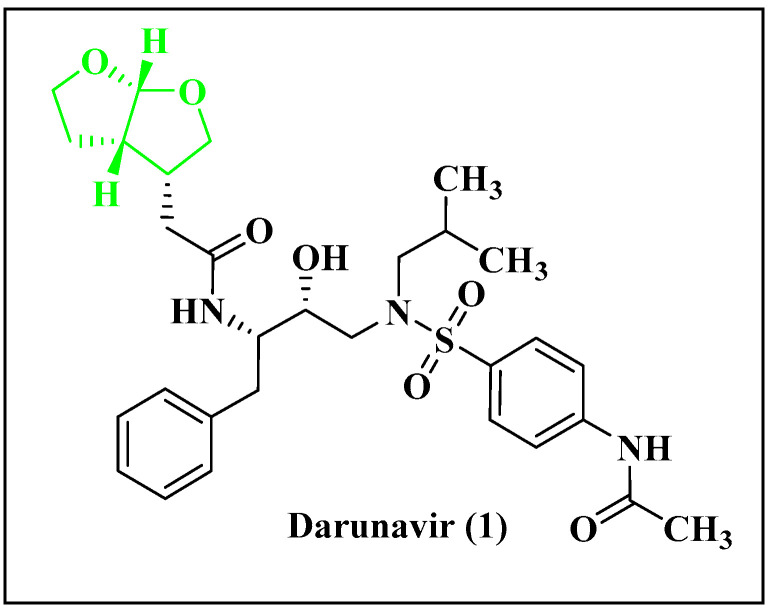
Chemical structure of darunavir (**1**).

Kate et al. have demonstrated the synthesis of darunavir (Figure 6), which is considered to be a protease inhibitor [112] and used in low doses for the treatment of HIV. Raltegravir [113] and stavudine [114] are the other available drugs which also show similar properties in this particular domain.

**Figure 7:**
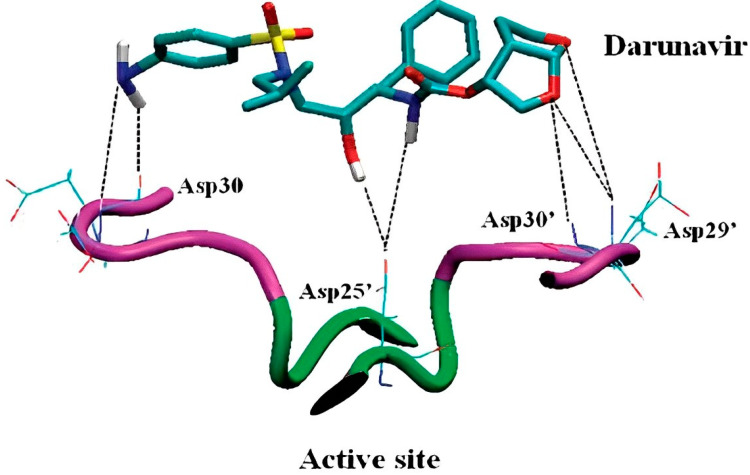
The interaction of darunavir with HIV-1 PR.

Darunavir becomes stabilized inside the cavity of the enzyme by making a hydrogen bonding interaction through the coordination of the hydroxyl group, 4-amino phenyl and tetrahydrofuran ring system with the active site (Asp25′) or near to the active site (Asp29/29′, Asp30/30′) of the amino acid residues as present in HIV-1 PR (Figure 7) [115].

**Scheme 1:**
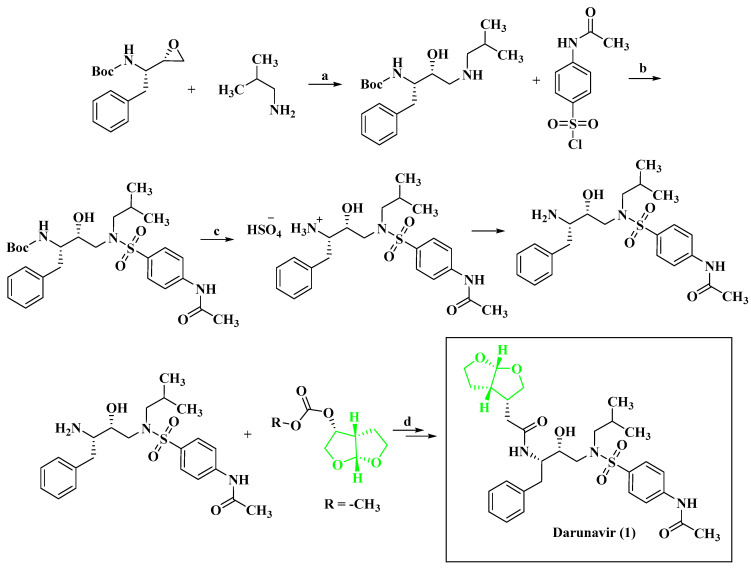
Synthesis of darunavir.

Key synthetic steps for Scheme 1 [116]: (a) ring opening of epoxide with primary amine; (b) N-protected 4-aminobenzenesulfonyl halide; (c) Boc deprotection; and (d) nucleophilic addition followed by separation of the product **1**.

Reagents and conditions: (a) A mixture of (2*S*,3*S*)-1,2-Epoxy-3-(Boc-amino)–4-phenylbutane and isobutyl amine was heated at 65–75 °C and (b) N-acetyl sulphanilyl chloride was added at 5–15 °C to the pre-cooled mixture of (1*S*,2*R*)-(1-Benzyl-2-hydroxy-3-(isobutyl-amino) propyl) carbamic acid tert-butyl ester in N, N-dimethylacetamide. Then, triethyl amine was added to the reaction mixture at a temperature below 30 °C and (c) Boc deprotection was done by taking the corresponding carbamic acid tert-butyl ester in isopropyl alcohol at 25–35 °C; after that, aqueous sulphuric acid solution was added to the reaction mixture at 25–35 °C. Then, the amino- N-((2*R*,3*S*)-3-amino-2-hydroxy-4-phenylbutyl)-N-isobutylbenzene sulfonamide sulphate salt was treated with potassium carbonate solution in water and 4-Amino-N-((2*R*,3*S*)-3-amino–2-hydroxy-4-phenylbutyl)-N–isobutylbenzenesulfonamide was obtained above in water. Then, to a stirred mixture of (d) potassium carbonate, isopropyl acetate and water, 4-amino-N-((2*R*,3*S*)-3-amino-2-hydroxy-4-phenylbutyl)-N-isobutyl benzene sulphonamide was added at 25–35 °C, and the reaction mixture was cooled to 15–25 °C; after that, (3*R*,3a*S*,6a*R*)-Hydroxyhexa hydrofuro [2,3-b] furanyl succinimidyl carbonate was added at 15–25 °C. After completion of the reaction, the crude reaction mixture was purified to obtain the final product **1**.

Structure–Activity Relationship of Darunavir: The structure–activity relationship of darunavir analogues is shown in Table 1, in which the incorporation of thiazole and ethyl phosphonate subunit as **R^1^**, along with benzo[d][1,3]dioxole moiety as **R^2^**, increases its activity significantly [117]. Darunavir and its corresponding synthetic analogues show a distinctive mechanism of action, as characterized by dual functionality. It works as HIV-1 protease inhibitor and also hinders the dimerization process of the HIV-1 protease [118]. Mostly, the Darunavir class of compounds exhibits a binding affinity towards plasma proteins such as alpha-1-acid glycoprotein (AAG or AGP) [119]. The CheckMate^TM^ Mammalian Two-Hybrid System was utilized to establish a dual luciferase assay. This assay was employed to assess the susceptibility of HIV-1_LAI_ to a variety of drugs and evaluate the cytotoxic effects of the drugs.

A 3-(4,5-dimethylthiazol-2-yl)-2,5-diphenyltetrazolium bromide assay was employed for determining drug susceptibility and cytotoxicity [120]. The chromogenic substrate Lys-Ala-Arg-Val-Nle-paranitro-Phe-Glu-Ala-Nle-amide was used to determine the kinetic parameters [121].

Mechanism of Action of Darunavir: Darunavir interacts with the protease enzyme of HIV-1 to prevent the dimerization and enhance the catalytic activity. As a result, the cleavage of the proteins is disturbed, and ultimately the replication of the virus is stopped, after the application of this drug to HIV-infected cells [122]. Generally, the interaction takes place with the primary chains of Asp-29 and Asp-30 amino acids present in the active site of the protease enzyme.

#### 5.1.2. Anti-HIV Agent Fluoroquinolone-Isatin-Thiosemicarbazone Hybrids

**Figure 8:**
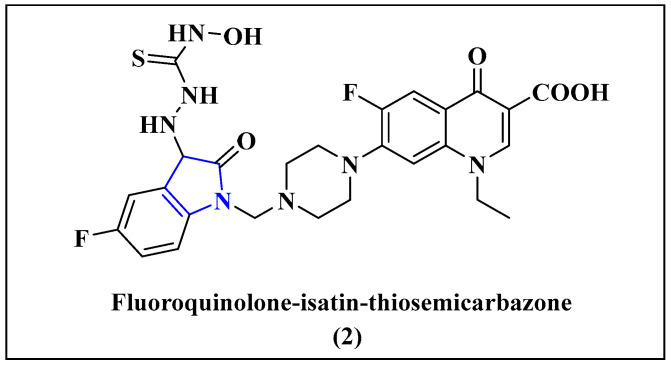
Fluoroquinolone-isatin-thiosemicarbazone hybrid (**2**), a potential anti-HIV agent.

The molecular-hybrid approach was introduced to understand the synergistic effects of the two compounds in order to generate a new structural entity with superior properties [123] for inhibiting the viral replication. Isatin derivatives have been shown to exhibit antiviral activity against a range of viruses, including HIV-1. Recently, hybrid fluoroquinolone-isatin derivatives have attracted attention due to their promising anti-HIV properties (Figure 8) [124]. 

**Scheme 2:**
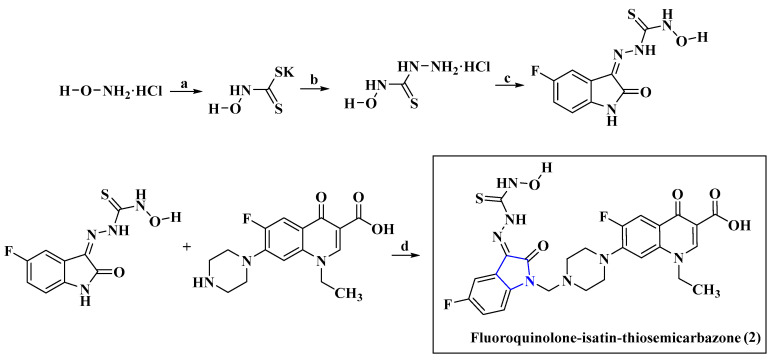
Synthesis of fluoroquinolone-isatin-thiosemicarbazone hybrid.

Key synthetic steps for Scheme 2 [125]: (a) A solution of *N*-hydroxylamine in absolute ethanol was added to potassium hydroxide and carbon disulphide, and the mixture was stirred at 0−5 °C to form the corresponding potassium salt of dithiocarbamates; (b) hydrazine hydrate was added to the reaction mixture and stirred at 80 °C; after completion of the reaction, it was cooled to 0 °C to obtain the corresponding thiosemicarbazide; (c) to a hot dispersion of thiosemicarbazide in ethanol was added an equimolar aqueous solution of sodium acetate, and to this solution a further equimolar ethanolic solution of 5-F-isatin was added, and the mixture was stirred while being heated on a hot plate for 4−15 min. The resultant precipitate was filtered off and dried. The product was recrystallized from 95% ethanol; (d) the *N*-Mannich bases were further synthesized by condensing the acidic imino group of isatin derivatives with formaldehyde and secondary amine (4-ethyl-7-fluoro-1-oxo-6-(piperazin-1-yl)-1,4-dihydronaphthalene-2-carboxylic acid) by irradiating the reaction vessel in a microwave reactor for 3−15 min at 455 W, followed by purification to obtain the corresponding product **2**.

Reagents and conditions: (a) CS_2_, KOH, C_2_H_5_OH, 0−5 °C; (b) NH_2_NH_2_.H_2_O, 80 °C, conc. HCl; (c) 5-F-Isatin, CH_3_COONa; (d) 30% HCHO, 455 W, 3−15 min.

Mechanism of Action of Fluoroquinolone-isatin-thiosemicarbazone: Isatinyl thiosemicarbazone derivatives have been found to exhibit anti-HIV activity by hindering the viral protease enzyme’s function. The viral protease enzyme plays a pivotal role in the maturation of the HIV virion by interrupting its activity; as a result, the production of the corresponding infectious virions stops. Isatinyl thiosemicarbazone derivatives interact with the active site of the viral protease enzyme, and hence its activity is stopped [126,127].

#### 5.1.3. Anti-HIV Agent Amprenavir

**Figure 9:**
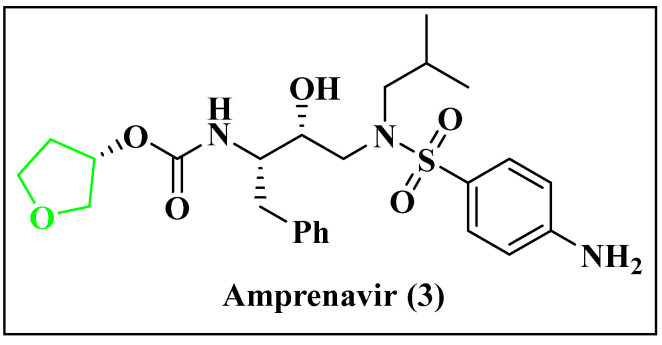
Chemical structure of amprenavir (**3**).

**Figure 10:**
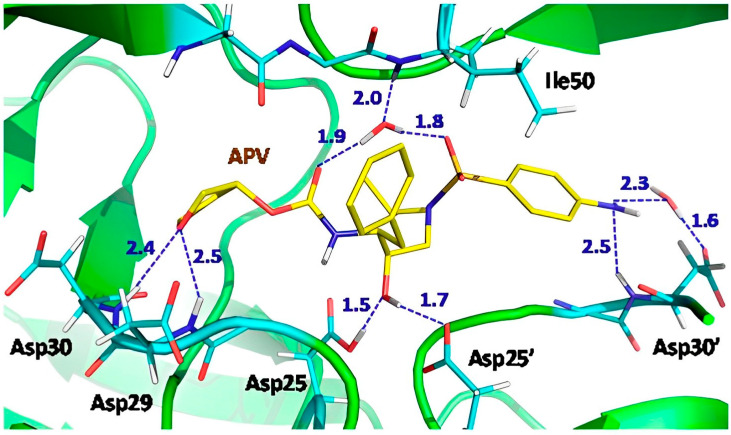
H-bonds and water-mediated interactions formed by amprenavir with the active site.

The drug amprenavir (Figure 9) is primarily used to treat HIV infections; it acts as a protease inhibitor. It binds to the active site of the enzyme and inhibits its activity. It prevents the cleavage of viral polyproteins, which leads to the development of immature non-infectious viruses [128]. Amprenavir’s hydroxyl group interacts with Asp25 and Asp25′ residues of the protein at the catalytic site. Along with that, there are stable H-bonding interactions between the hydroxyl group of the drug with the catalytic site of the aspartic acid side chains (Figure 10) [129].

**Scheme 3:**
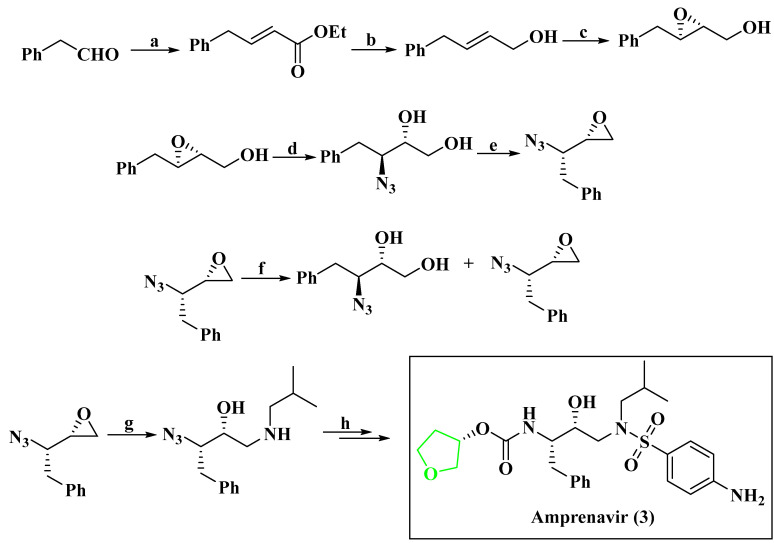
Synthesis of amprenavir.

Key synthetic steps for Scheme 3 [130]: (a) Formation of chalcone by the reaction of 2-phenylacetaldehyde and Ph_3_PCHCO_2_Et at 90 °C in toluene; (b) reduction of the ester by lithium aluminium hydride and AlCl_3_ in diethyl ether; (c) chiral epoxidation; (d) epoxide ring opening, followed by (e) epoxide ring closing and further(f) ring opening; and formation of gem-diol derivatives which formed product **3** by reacting with (g) isobutyl amine and (h) N-hydroxysuccinimidyl carbonate of (S)-3-hydroxytetrahydrofuran.

Reagents and conditions: (a) Ph_3_PCHCO_2_Et, PhH, 90 °C; (b) LiAlH_4_, AlCl_3_ (30 mol %), Et_2_O, 0 °C; (c) mCPBA, CH_2_Cl_2_, 0 °C; (d) Ti(O^i^Pr)_4_, TMSN_3_, C_6_H_6_, 70 °C; (e) p-TsCl, Bu_2_SnO (2 mol %), Et_3_N, DMAP (10 mol %), CH_2_Cl_2_, 0 °C; (f) K_2_CO_3_, MeOH, 0 °C; (g) (S,S)-Co(salen)OAc (0.5 mol %), THF, H_2_O (0.5 equiv), 25 °C; (h) (1) ^i^BuNH_2_, ^i^PrOH, 50 °C, (2) PPh_3_, H_2_O, THF, 25 °C; (2) N-hydroxysuccinimidyl carbonate of (S)-3-hydroxytetrahydrofuran, Et_3_N, CH_2_Cl_2_, 25 °C.

Structure–Activity Relationship of Amprenavir: It shows antiviral properties against the HIV-1 virus in an in vitro study against the C8166 cell line [131]. The efficacy of HIV-1 protease inhibitors was assessed using the fluorescence resonance energy transfer (FRET) technique. A specific protease substrate [Arg-Glu(EDANS)-Ser-Gln-Asn-Tyr-Pro-Ile-Val-Gln-Lys(DABCYL)-Arg], was employed. The determination of the inhibitor’s binding dissociation constant (K_i_) involved fitting the initial velocity plot against inhibitor concentrations to the Morrison equation through non-linear regression analysis [132]. The EC_50_ values were compared with those of amprenavir, resulting in the conclusion that the biaryl subunit with varying substituents showed lower efficiency. However, compounds that featured substituents of –NH_2_ for **R^1^** and 3-pyridyl or 4-pyridyl for **R^2^** exhibited increased solubility and stronger enzyme inhibitory activity at a sub-nanomolar level. These compounds were found to be 2–10 times more active than amprenavir, as described in Table 2 [133].

Mechanism of Action of Amprenavir: Amprenavir binds to the active site of the protease and inhibits the activity of the enzyme. This inhibition prevents the cleavage of the gag-pol polyprotein. Amprenavir competes with the natural substrate of the viral protease enzyme, which is a precursor protein of the viral genome. This competition leads to the formation of a stable complex between the drug and the enzyme, preventing the enzyme from cleaving the protein. As a result, non-infectious viral particles are produced, leading to a reduction in the number of viral particles in the body [134]. In Table 3 other marketed anti-HIV drugs are listed with their mechanism of action, ways of use and side effects.

Some other synthesized compounds that show activity against HIV are given in Table 4.

### 5.2. Anti-HCV Agent

#### 5.2.1. Anti-HCV Agent Asunaprevir

**Figure 11:**
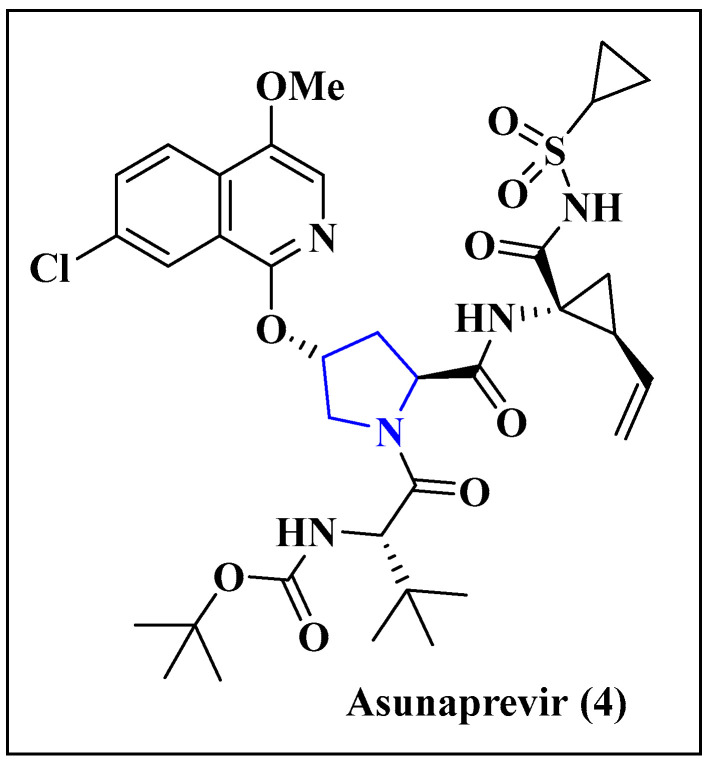
Chemical structure of asunaprevir (**4**).

**Figure 12:**
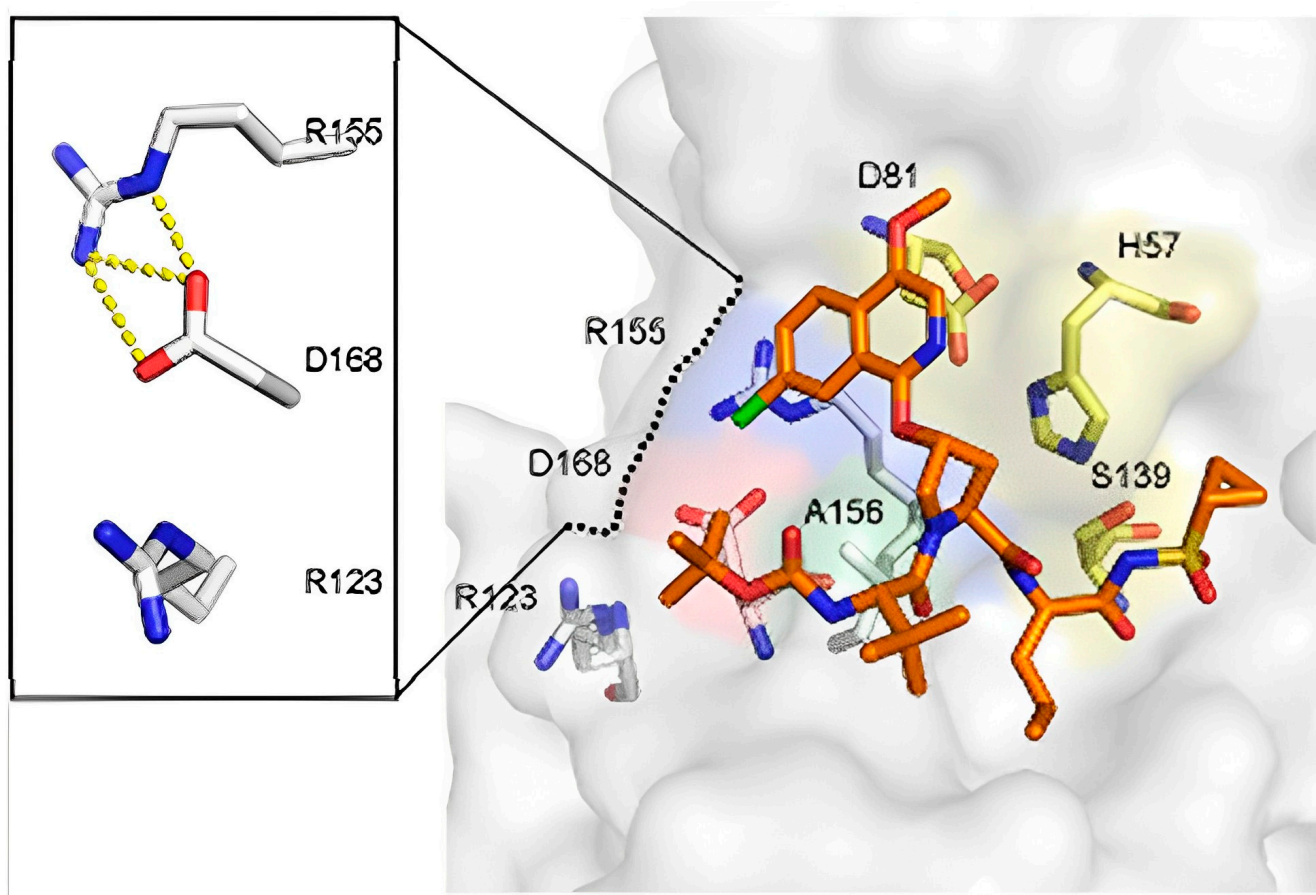
Structure of HCV NS3/4A protease in complex with asunaprevir.

Asunaprevir (Figure 11) is an orally efficacious NS3 protease inhibitor used for the treatment of hepatitis C virus infection. This tripeptidic acyl sulfonamide is an inhibitor of the enzyme NS3/4A, and is now in phase III clinical trials for the treatment of hepatitis C virus infection. The activity of asunaprevir showed a robust antiviral response in early clinical trials. Suzuki et al. have studied the antiviral activity and toxicological profile of asunaprevir [147]. It inhibits the activity of proteases by binding to the active site. Viral polyproteins cannot be cleaved by this inhibition, which produces undeveloped and non-infectious viral particles (Figure 12) [148].

**Scheme 4:**
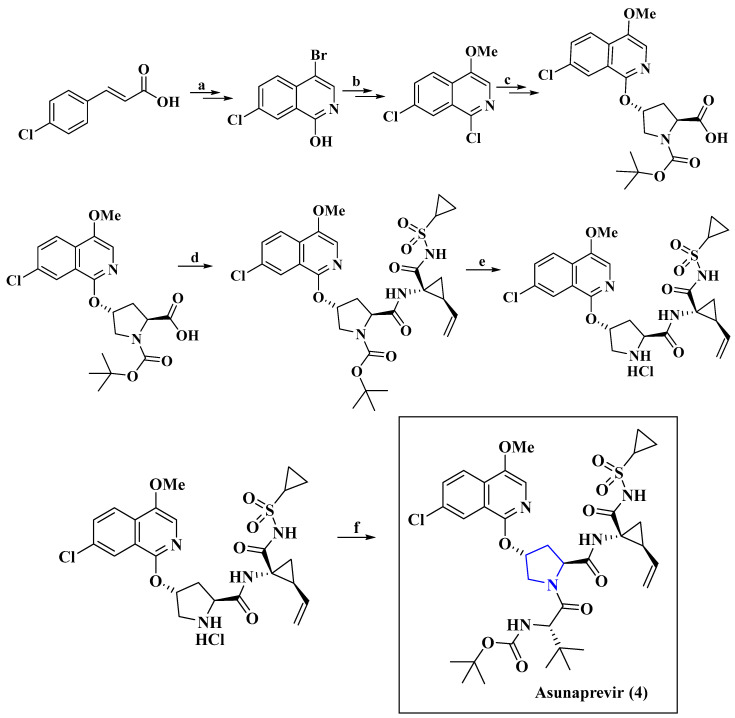
Synthesis of anti-HCV agent asunaprevir.

Key synthetic steps for the Scheme 4 [149]: (a,b) (*E*)-3-(4-chlorophenyl)acrylic acid is cyclized, and the derivatization (c) reacts with *N*-Boc-3-(*R*)-hydroxy-L-proline via nucleophilic addition (d) by peptide coupling reaction. The synthesized fragment reacts with (1*R*,2*S*)-1-amino-*N*-(cyclopropylsulfonyl)-2-vinylcyclopropanecarboxamide (TsOH salt), followed by (e) deprotection of proline nitrogen. (f) The final product **4** was obtained by the peptide coupling reaction with *N*-Boc-t-butyl-L-glycine.

Reagents and conditions: (a) (i) DPPA, Et_3_N, benzene, rt; (ii) Ph_2_CH_2_, reflux; (iii) NBS, MeCN, reflux. (b) (i) POCl_3_, reflux; (ii) (1) n-Bu-Li, THF, −78 °C, (2) (*i*-PrO)_3_B, −78 °C; (3) 50% H_2_O_2_, Na_2_SO_3_ −78 °C; (iii) MeOH, MeCN, TMSCHN_2_, 0 °C–rt. (c) *N*-Boc-3-(*R*)-hydroxy-L-proline, t-BuOK, DMSO, 10 °C. (d) HATU, Hunig’s base *i*-Pr_2_Net, (1*R*,2*S*)-1-amino-*N*-(cyclopropylsulfonyl)-2-vinylcyclopropanecarboxamide (TsOH salt), rt; (e) HCl (conc.), MeOH, reflux; (f) HATU, Hunig’s base, *N*-Boc-t-butyl-L-glycine, DCM, 0 °C–rt.

Structure–Activity Relationship of Asunaprevir: The structure-activity relationship studies show that the incorporation of a hydroxyl group at the **R^1^** position decreases its antiviral activity (Table 5). The isoquinoline series with methoxy and chlorinated analogues proved to be potent inhibitors of the NS3/4A protease (GT-1a NS3/4A enzyme) which extended to excellent inhibitory activity in the replicon at the **R^2^** substituent. The antiviral activity of the drug was evaluated through a two-part study. Initially, a single ascending dose (SAD) study was conducted in patients infected with genotype 1, followed by a subsequent multiple ascending dose (MAD) study [149].

Mechanism of Action of Asunaprevir: Asunaprevir is highly active against HCV NS3 protease [150], which is responsible for processing the HCV polyprotein into individual viral proteins. The production of new viral proteins is prevented with the use of this drug compound, and as a result, the progression of HCV-related liver disease is reduced. It is typically used in combination with other antiviral drugs to enhance efficacy and minimize drug resistance [52].

#### 5.2.2. Anti-HCV Agent 3-(1,2,4-oxadiazole)-quinolone

**Figure 13:**
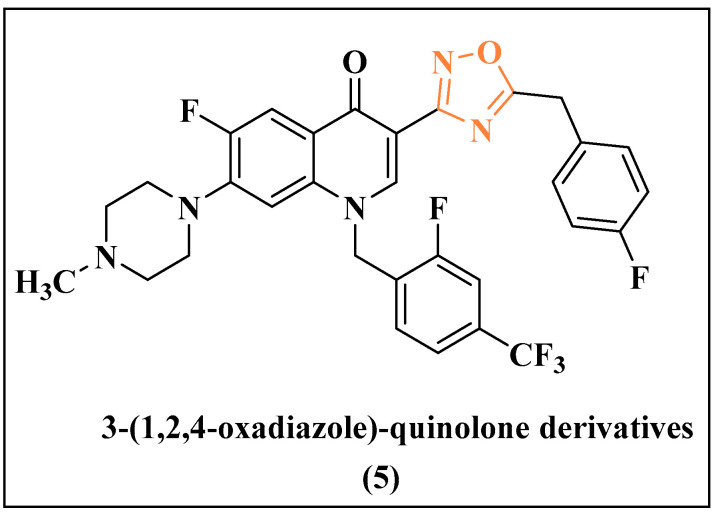
3-(1,2,4-oxadiazole)-quinolone derivative (**5**) with anti-HCV activities.

Studies have shown that 3-heterocyclic quinolones (Figure 13) can inhibit NS5B polymerase activity by binding to an allosteric site. This binding triggers a change in the protein’s structure, resulting in the inhibition of RNA replication. In vitro and in vivo studies have demonstrated the significant antiviral activity of these compounds against HCV and indicated their roles as promising lead candidates for further development [151].

**Scheme 5:**
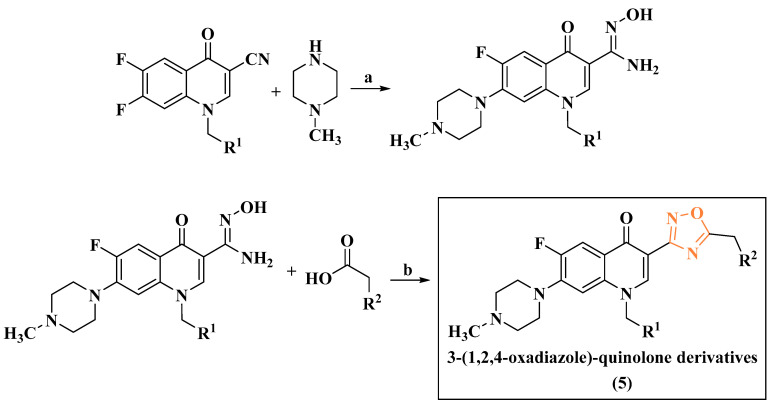
Synthesis of 3-(1,2,4-oxadiazole)-quinolone derivatives.

Key synthetic steps for Scheme 5 [151]: (a) The nitrile moiety of quinolone is converted to the intermediate hydroxyamidine through reaction with hydroxyl amine; (b) the resulting hydroxyamidine intermediates are converted to a variety of 1,2,4-oxadiazole target compounds by reacting with appropriate carboxylic acids to obtain product **5**.

Reagents and conditions: (a) CH_3_CN, reflux (b) NH_2_OH, DIEA, aq. EtOH; (c) HBTU, DIPEA, 200 °C, microwave.

Structure–Activity Relationship of 3-(1,2,4-oxadiazole)-quinolone derivatives: The activity (IC_50_) against the NS5B polymerase enzyme (Table 6) was determined by scintillation proximity assays (SPA) [152]. For a high-throughput screening (HTS) campaign to identify inhibitors of NS5B polymerase, a scintillation proximity assay (SPA) format was employed. Scintillation proximity assays (SPAs) are an efficient technique that can be used to detect enzymes, receptors, radioimmunoassays and molecular interac-tions. This assay format allowed for the screening of compounds that effectively inhibited the enzymatic function of NS5B polymerase, specifically targeting the wild-type (genotype 1b) enzyme. The IC_50_ values of the compounds were assessed against NS5B polymerase, and their efficacy was determined in using a cell-based viral replication surrogate assay called the replicon system [152]. **R^1^** and **R^2^** mainly stabilize the compounds in the hydrophobic pockets. The inhibition activity of these two functionalities present in the quinolone moiety is shown in Table 6 [153]. It is clearly found that the presence of the –F or –CF_3_ group in either **R^1^** or **R^2^** is very much responsible for modulating the corresponding activity [153].

Mechanism of Action of 3-(1,2,4-oxadiazole)-quinolone derivatives: 3-(1,2,4-oxadiazole)-quinolone derivatives show promising inhibitory activity against HCV NS5B polymerase and NS3 protease. Cyclophilin, a protein present in the host cell of HCV, also is affected by the interaction of such heterocyclic compounds; consequently, the replication process of this virus becomes affected. By targeting multiple stages of the HCV life-cycle, these compounds can reduce the amount of virus and slow or stop the progression of HCV-related liver diseases [154].

#### 5.2.3. Anti-HCV Agent Grazoprevir

**Figure 14:**
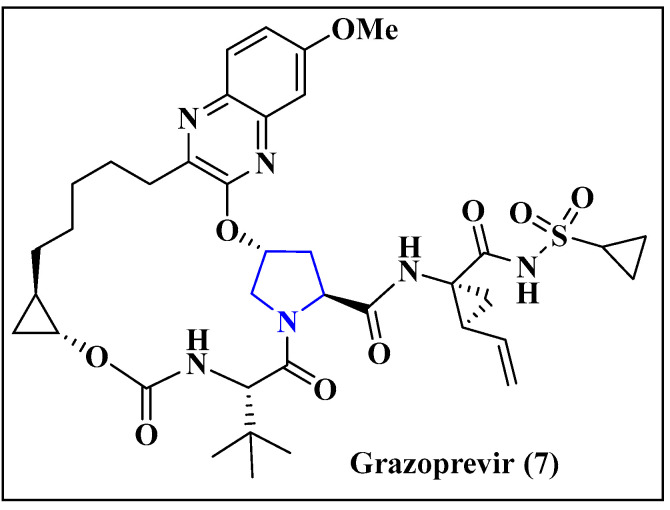
Chemical structure of grazoprevir (**7**).

Grazoprevir (Figure 14) is a potent inhibitor of RNA synthesis in HCV (due to the action of two different DAAs as NS5A and NS3/4A inhibitors), representing high genetic barriers to resistance. The mechanism of action and pharmacodynamic properties, as well as the pharmacokinetics, clinical uses, safety and efficacy of elbasvir/grazoprevir in managing a large variety of conditions, including cases in the presence of cirrhosis, co-infection with HIV and patients having inherited blood disorders, were nicely reviewed by Kassas et al. [155]. Sofosbuvir [156], ledipasvir [157] and telaprevir [158] are the reported anti-HCV drugs with similar synthetic procedures.

**Scheme 6:**
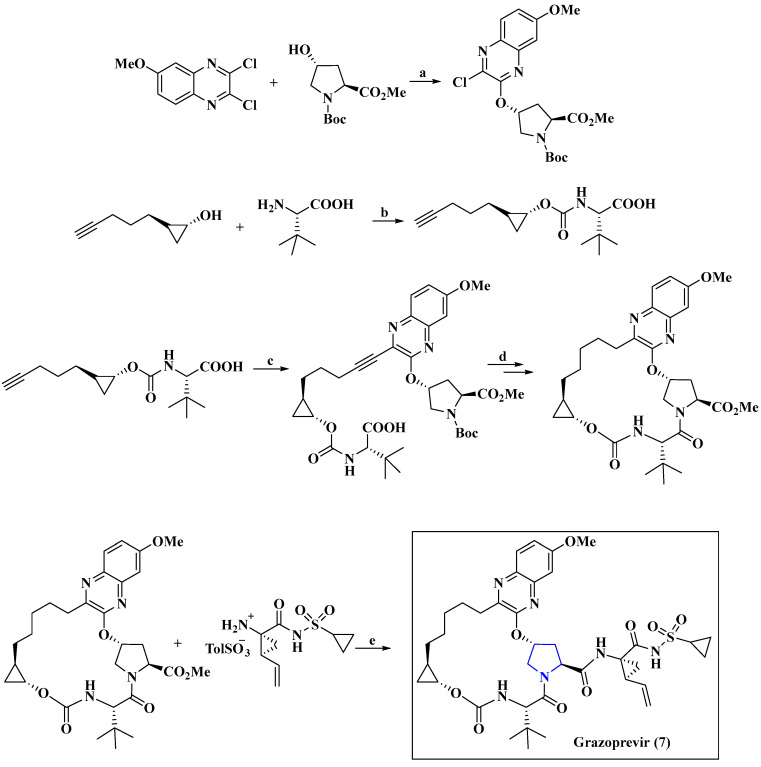
Synthesis of grazoprevir.

Key synthetic steps for Scheme 6 [159]: Grazoprevir was synthesized starting from the (a) cross coupling strategy of 2,3-dichloro-6-methoxyquinoxaline with substituted proline derivative to obtain the corresponding five membered heterocyclic core followed by (b) esterification of the (1*R*,2*R*)-2-(pent-4-yn-1-yl)cyclopropan-1-ol with (*S*)-2-amino-3,3-dimethylbutanoic acid and (c) metal catalyzed coupling reactions of the fragments, followed by (d) cyclization through intramolecular peptide coupling (e). The final product **7** was obtained by the peptide coupling with the allylic sulfonamide, as shown in Scheme 6.

Reagents and conditions: (a) DBU (1.05 equiv.), DMAc, 50 °C; (b) CDI, Hunig’s base, 95 °C, 2.5 h; (c) Pb(OAc)_2_, P(*t*-Bu)_3_BF_4_, K_2_CO_3_, CPME/MeCN; (d) (1) Pd/C, H_2_, IPAc/MeOH; (2) (i) PhSO_3_H, (ii) HATU, NEt_3_, MeCN; (e) DEC, pyridine, MeCN.

Structure–Activity Relationship of Grazoprevir: A group of linear HCV NS3/4A protease inhibitors was created by removing the macrocyclic linker found in grazoprevir. This allows for the exploration of diverse quinoxalines while conferring conformational flexibility. Inhibitors with small substituents at the 3-position (**R^1^**) of quinoxaline were found to be effective in maintaining potency. The 3-chloroquinoxaline demonstrated outstanding potency against wild-type HCV NS3/4A protease. Replacing the cyclopropyl-sulfonamide with a more hydrophobic 1-methyl cyclopropyl-sulfonamide group (**R^2^**) generally enhances the potency of the resulting analogues. Similarly, substituting the tert-butyl group (**R^3^**) with a bulkier cyclopentyl moiety led to the development of compounds with improved potency (Table 7) [160]. The enzyme inhibition constants (K_i_) were determined for the wild-type genotype 1a NS3/4A protease, as well as the resistant variants R155K and D168A. Additionally, a subset of compounds underwent testing to determine their cellular antiviral potencies (EC_50_) using replicon-based antiviral assays. These assays, which assessed the efficacy of the compounds, were not only run against the wild-type HCV strain but also against the drug-resistant variants R155K, A156T, D168A, and D168V [161].

Mechanism of Action of Grazoprevir: Grazoprevir is a potent and selective inhibitor of the NS3/4A protease enzyme in the hepatitis C virus (HCV). The NS3/4A protease enzyme plays a critical role in HCV replication by cleaving the HCV polyprotein into the individual functional proteins necessary for the virus to replicate and propagate. Grazoprevir’s mechanism of action has been extensively studied and documented. Grazoprevir effectively inhibited the NS3/4A protease enzyme by binding to the enzyme’s active site, thereby preventing the cleavage of the HCV polyprotein and inhibiting HCV replication [162]. There are other drugs available on the market for the treatment of HCV, such as boceprevir, sofosbuvir, etc. Their mechanisms of action, ways of use and side effects are given in Table 8**.**

Some other synthesized compounds that show activity against HCV are given in Table 9.

### 5.3. Anti-HBV Agent

#### 5.3.1. Anti-HBV Agent Lamivudine

**Figure 15:**
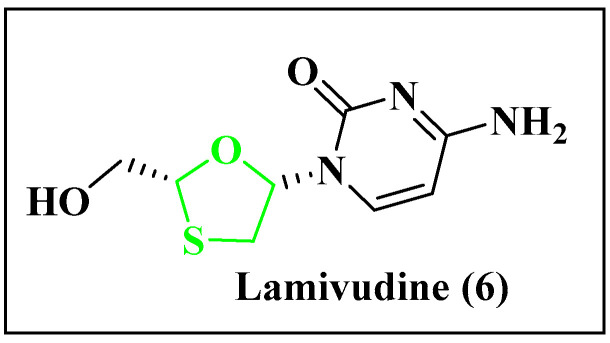
Chemical structure of lamivudine (**6**).

Lamivudine (Figure 15) is a nucleoside reverse transcriptase inhibitor that inhibits the reverse transcriptase of the human hepatitis B virus (HBV). It is a safe medicine with minimal side effects and can be prescribed for pregnant women and children over five years of age [172].

**Scheme 7:**
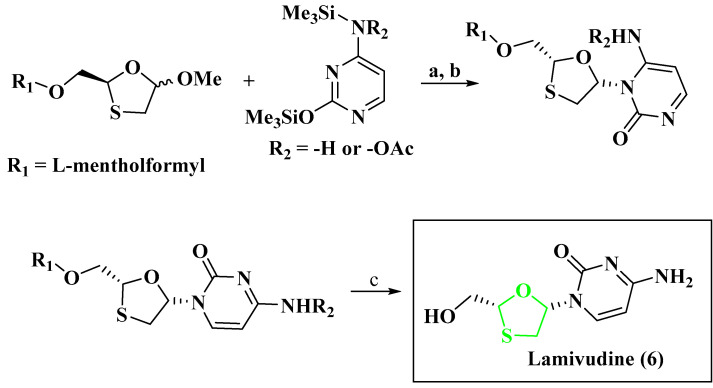
Synthesis of anti-HBV agent lamivudine.

Key synthetic steps for Scheme 7 [173,174]: Formation of mixture of diastereomer at 0 °C (b) separation of diastereomers is done by recrystallization, (c) diastereo pure compound is treated with methanolic K_2_CO_3_ to obtain product **6**.

Reagents and conditions: (a) Reactants are mixed and cooled to 0 °C in MeOH; (b) separation of diastereomers; (c) MeOH, K_2_CO_3_.

Mechanism of Action of Lamivudine: Lamivudine is a nucleoside analogue that is used in the treatment of hepatitis B virus (HBV) infection. The mechanism of action of lamivudine is based on its ability to inhibit HBV reverse transcriptase, which is a critical enzyme for viral replication. Once inside the infected cell, lamivudine is phosphorylated by cellular enzymes into its active triphosphate form. This active form of lamivudine competes with the natural nucleotide building blocks for incorporation into the growing viral DNA chain. However, lamivudine lacks the 3′-OH group required for further chain extension, thereby resulting in the termination of viral DNA synthesis [175].

#### 5.3.2. Anti-HBV Agent Entecavir

**Figure 16:**
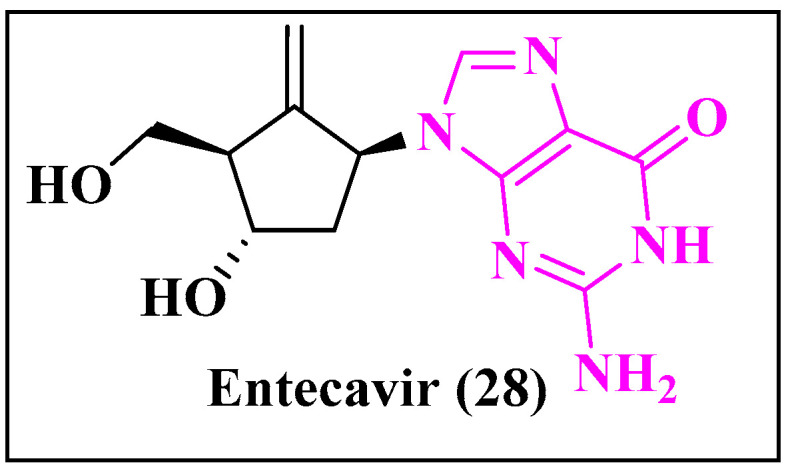
Chemical structure of entecavir (**28**).

**Figure 17:**
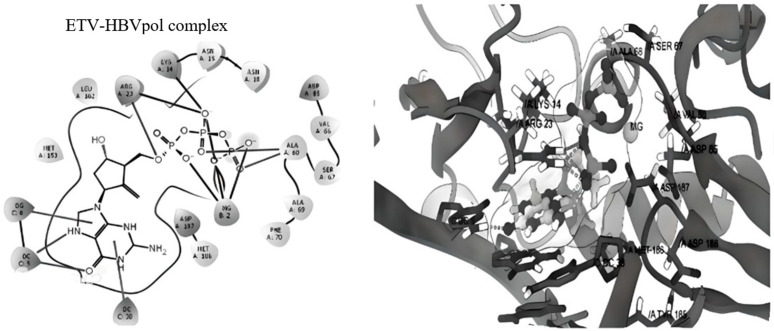
Entecavir triphosphate docking with HBVpol.

Entecavir (Figure 16) is a guanosine nucleoside analogue active against hepatitis B (HBV). It is highly efficient in preventing all stages of replication. Compared to the other Hepatitis B drugs, lamivudine and entecavir are more effective; the corresponding triphosphate binds with HBVpol with amino acid residues ARG A: 23, LYS A: 14, ASN A: 18 and ALA A: 68 and effectively inhibits its activity (Figure 17) [176,177].

**Scheme 8:**
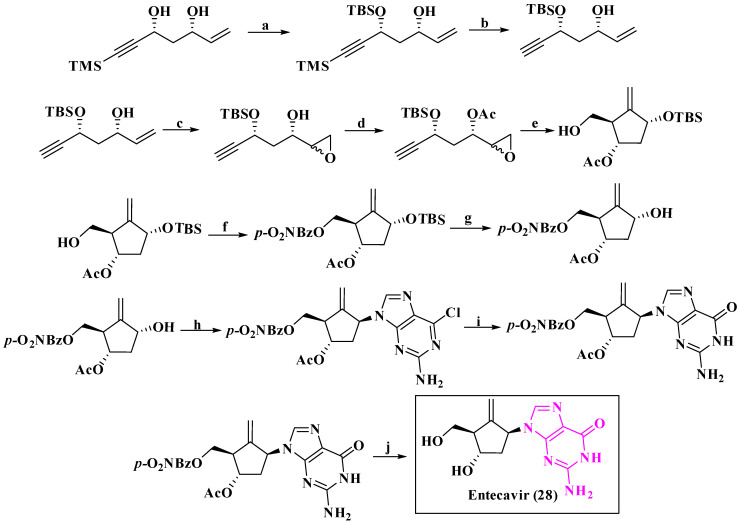
Synthesis of entecavir.

Key synthetic steps for Scheme 8 [178]: (a) Protection of aliphatic alcohol; (b) activation of the terminal alkyne; (c,d) synthesis of the epoxide followed by (e) intramolecular cyclization; (f,g) protection and deprotection of the alcohols; (h) Mitsunobu reaction with 2-amino-6-chloropurine; followed by (i) acid treatment; and (j) saponification to obtain the Entecavir **28**.

Reagents and conditions: (a) TBSCI (1.1 equiv.), imidazole, THF, rt; (b) K_2_CO_3_ cat., MeOH; (c) m-CPBA, CH_2_Cl_2_; (d) Ac_2_O, NEt_3_, DMAP cat., CH_2_Cl_2_; (e) Cp_2_TiCl_2_ 20 mol%, IrCl(CO)(PPh_3_)_2_ 10 mol%, Mn (2 equiv.), collidine, TMSCl, H_2_ (4 bar), THF; (f) p-O_2_NBzCl, NEt_3_, CH_2_Cl_2_; (g) 5% (+)-CSA, MeOH; (h) 2-amino-6-chloropurine, DIAD, PPh_3_, THF, −10 °C; (i) HCOOH, 50 °C; (j) MeONa, MeOH.

Structure–Activity Relationship of Entecavir: An extensive investigation of the structure–activity relationship (SAR) of entecavir and its analogues is shown in Table 10**.** It was discovered that these compounds are the most potent inhibitors of HBV replication, with the ability to effectively inhibit lamivudine-resistant HBV. These compounds are carbocyclic guanosine nucleoside analogues (**R^1^**) and are highly effective when tested against HBV in HepG2.2.15 cells [179]. The plasma half-life of entecavir in rats and dogs is 4–9 h. It is metabolized by HepG2 cells to the corresponding mono-, di-, and triphosphates.

Mechanism of Action of Entecavir: Entecavir is a nucleoside analogue that inhibits hepatitis B virus (HBV) DNA replication by interfering with the activity of viral polymerase, an enzyme essential for the virus to replicate its genetic material. In HBV-infected cells, it is phosphorylated into its active form, entecavir triphosphate, which competes with the natural substrate, deoxyguanosine triphosphate, for incorporation into the elongating viral DNA chain. The incorporation of entecavir triphosphate into the viral DNA chain leads to chain termination, preventing further extension of the viral DNA and ultimately inhibiting HBV replication. Entecavir’s mechanism of action has been extensively studied and has been shown to be highly effective in suppressing HBV replication and reducing liver damage. Due to its high potency and low risk of developing viral resistance, entecavir has become one of the preferred first-line treatments for chronic HBV infection [180,181].

#### 5.3.3. Anti-HBV Agent Dehydro-Andrographolide and Andrographolide Derivatives

Dehydro-andrographolide and andrographolide compounds have demonstrated the ability to inhibit the replication of HBV DNA, with IC_50_ values of 22.58 and 54.07 μM, respectively [172].

**Scheme 9:**
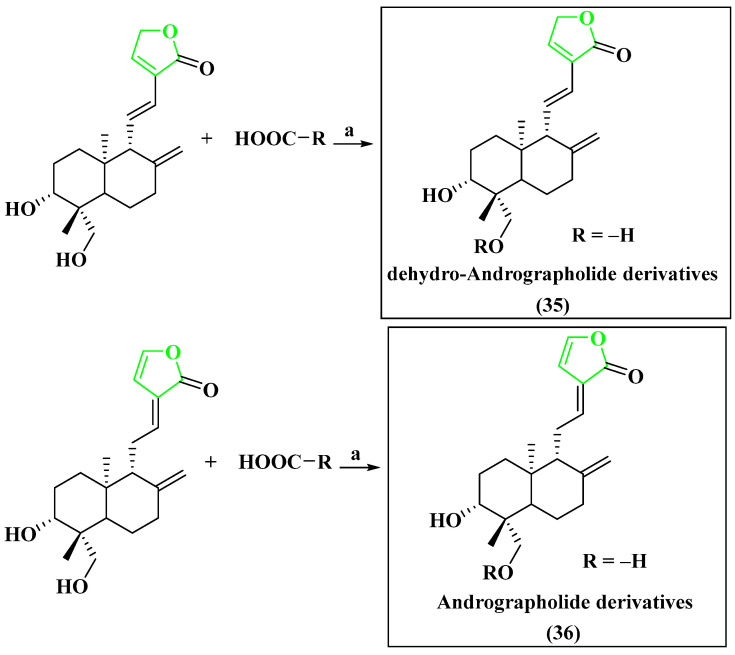
Synthesis of dehydro-andrographolide (**35**) and andrographolide (**36**) derivatives.

Key synthetic steps for Scheme 9 [172]: (a) Compounds are obtained with the help of esterification reaction with acids in the presence of 4-dimethylaminopyridine (DMAP) and N′,N′-dicyclohexylcarbodiimide (DCC) in anhydrous dichloromethane to obtain the product **35**.

Reagents and conditions: (a) corresponding acids, DMAP, DCC, CH_2_Cl_2_, rt.

Structure–Activity Relationship of dehydro-Andrographolide: The SARs of the derivatives indicate that having a free hydroxyl group at C-2 can result in enhanced anti-HBV properties. Additionally, maintaining the double bond between C-8 and C-17, as well as the conjugated double bonds between C-11 and C-14, or C-12 and C-15, is crucial for preserving anti-HBV activity and decreasing cytotoxicity. To improve the anti-HBV activity, it is useful to incorporate the 3-methoxycinnamoyl, nicotinoyl, 2-furoyl, or 2-thenoyl groups shown in Table 11 [105]. The anti-HBV activity of the compounds derived from dehydro-andrographolide and andrographolide were investigated. Specifically, their ability to inhibit the secretion of HBsAg and HBeAg, as well as HBV DNA replication, was evaluated using HepG 2.2.15 cells in vitro. Tenofovir, a known antiviral agent, was used as the positive control in the study [182].

Mechanism of Action of dehydro-Andrographolide: Dehydro-andrographolide inhibits HBV replication by blocking the binding of the HBV core protein to viral RNA. It also activates the host immune response, which can help control HBV replication and clear infected cells [183]. Overall, dehydro-andrographolide has shown promising anti-HBV activity through its ability to inhibit viral replication and enhance the host immune response. However, further studies are needed to fully understand its mechanisms of action and potential clinical applications [184]. The details of other anti-HBV drugs, along with their mechanisms of action, are mentioned in Table 12.

Some other synthesized compounds with activity against HBV are tabulated below (Table 13).

Capsid assembly modulators (CpAMs) belong to a novel category of antiviral compounds that specifically target the core protein of the hepatitis B virus (HBV) to interfere with the assembly process. HepG2.2.15 cells are a type of human hepatoblastoma cells that have been genetically modified to stably express the hepatitis B virus (HBV). The compounds under investigation inhibit HBV replication by interfering with the assembly of the HBV capsid protein [188].

**Table 13:** Synthesized anti-HBV compounds.

Sl. No.	Antiviral Agent	Drug Target	Activity
1.	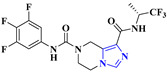	HBV [189]	Capsid assembly modulators (CpAMs) are antiviral compounds that target the core protein of the hepatitis B virus (HBV) to disrupt assembly. In HepG2.2.15 cells, which express HBV, these compounds inhibit HBV replication by interfering with capsid protein assembly with EC_50_ = 511 nM.
2.	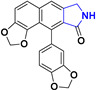	HBV [190]	The anti-HCV activities were tested in the Huh-Luc/neo cell line and cytotoxicity of the test compound was determined on both MT-2 cell lines with EC_50_ = 10 µM.

### 5.4. Anti-RSV Agent

#### Anti-RSV Agent Ribavirin

**Figure 18:**
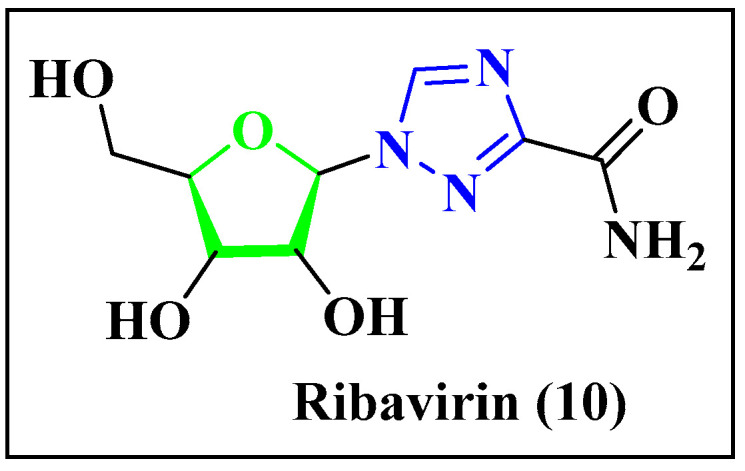
Chemical structure of ribavirin (**10**).

The antiviral property of this drug (Figure 18) was studied in 1972 [191]. This drug is presently used for the treatment of RSV. DeVincenzo et al. have studied the inhibitory activities of ribavirin against the RSV F-protein [192].

**Scheme 10:**
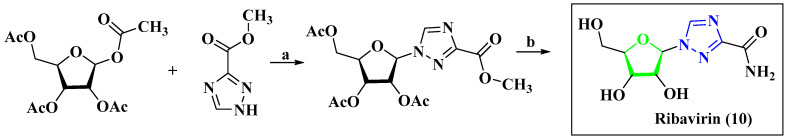
Synthesis of ribavirin.

Key synthetic step for the Scheme 10 [193]: (a) Nucleophilic substitution in the presence of bio-catalyst, (b) amide formation in the presence of NH_3_ to obtain product **10**.

Reagents and conditions: (a) Purine nucleoside phosphorylase, buffer solution of pH = 7; (b) NH_3_, MeOH.

Mechanism of Action of Ribavirin: Ribavirin is a broad-spectrum antiviral agent that has activity against a range of RNA and DNA viruses, including respiratory syncytial virus (RSV). The mechanism of action of ribavirin is not completely understood, but it is believed to involve several different mechanisms [194]. One proposed mechanism is that ribavirin interferes with the synthesis of viral RNA by inhibiting the activity of the viral RNA-dependent RNA polymerase. Another proposed mechanism is that ribavirin induces mutations in the viral genome, leading to non-functional or less-virulent viral particles. Additionally, ribavirin has been shown to stimulate the host’s immune response, which may contribute to its antiviral effects [195,196,197]. RSV-IGIV and palivizumab are the available drugs on the market for the treatment of RSV. Their mechanisms of action, ways of use and side effects are given in Table 14.

Some other synthesized compounds that show activity against RSV are given in Table 15.

### 5.5. Anti-HCMV Agent

#### 5.5.1. Anti-HCMV Agent Iso-Valganciclovir Hydrochloride

**Figure 19:**
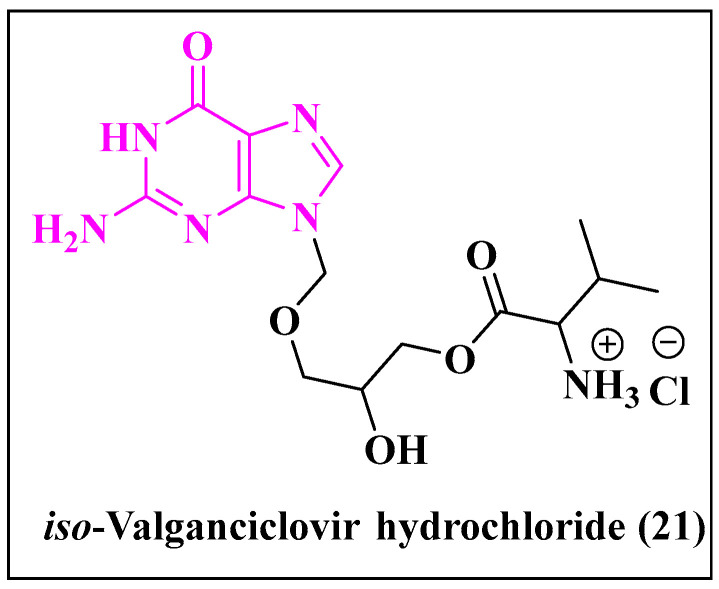
Chemical structure of iso-Valganciclovir hydrochloride (**21**).

Iso-Valganciclovir hydrochloride (Figure 19) is used for the treatment of cytomegalovirus (CMV). It is a type of nucleoside analogue and is the cutting-edge drug candidate against CMV [203].

**Scheme 11:**
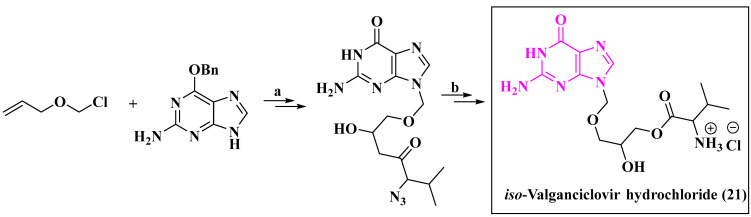
Synthesis of *iso*-valganciclovir hydrochloride.

Key synthetic step for Scheme 11 [204]: (a) Addition of reaction of 3-(chloromethoxy)prop-1-ene to *o*-benzyl guanine in the presence of a base, followed by oxidation; (b) addition reaction with *S*-2-azido-3-methylbutanoic acid and further reduction gives the final product **21,** as shown in Scheme 11.

Reagents and conditions: (a) (i) NaH, DMF; (ii) KMnO_4_, acetone; (ii) 10% Pd/C, MeOH; (iv) (*S*)-2-azido-3-methylbutanoic acid, DCC, DMSO; (b) 10% Pd/C, MeOH.

Mechanism of Action of *Iso*-Valganciclovir Hydrochloride: *Iso*-Valganciclovir hydrochloride is a prodrug of the antiviral agent ganciclovir, which is converted to its active form by hydrolysis of the valine ester in the liver and blood. The active form of ganciclovir works by inhibiting the viral DNA polymerase, which is essential for the replication of HCMV. By inhibiting the viral DNA polymerase, ganciclovir prevents the formation of new viral DNA chains, which ultimately inhibits HCMV replication [205].

#### 5.5.2. Anti-HCMV Agent Ganciclovir

**Figure 20:**
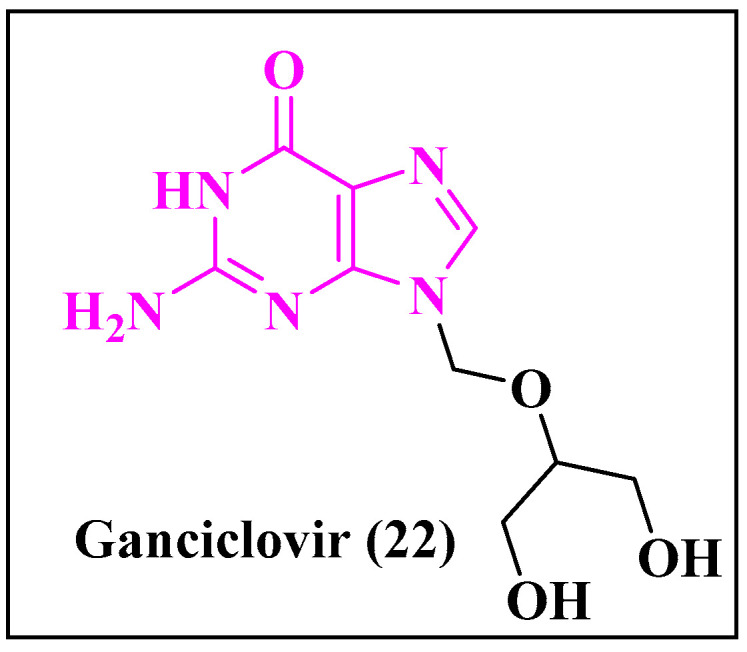
Chemical structure of ganciclovir (**22**).

Ganciclovir (Figure 20) is a marketed drug for the treatment of HCMV; it acts as a DNA polymerase to inhibit synthesis of viral DNA [206].

**Scheme 12:**
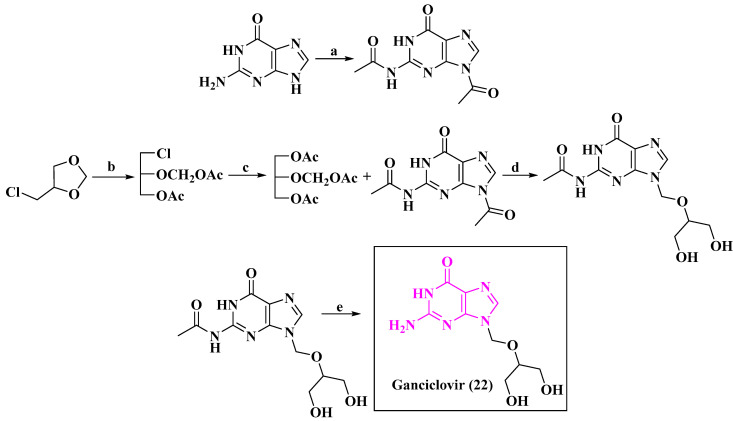
Synthesis of Ganciclovir.

Key synthetic steps for Scheme 12 [207]: (a) *N*-Acylation of the guanine then (d) reacts with 2-(acetoxymethoxy)propane-1,3-diyl diacetate to form N-(9-(((1,3-dihydroxypropan-2-yl)oxy)methyl)-6-oxo-6,9-dihydro-1H-purin-2-yl)acetamide (prepared from 4-(chloromethyl)-1,3-dioxolane) (e) by the deprotection of the amine and alcohol group; the final product **22** was thereby obtained.

Reagents and conditions: (a) Ac_2_O/HOAc, 140 °C; (b) Ac_2_O/HOAc/ZnCl_2_, r.t.; (c) KOAc/DMF, 150 °C; (d) EtSO_3_H, 165–170 °C; (e) 40% aq. MeNH_2_, 75 °C.

Mechanism of Action of Ganciclovir: Ganciclovir is an antiviral drug used to treat HCMV infections. It stops viral DNA synthesis by acting as a chain terminator, which inhibits the elongation of the viral DNA strand. Ganciclovir triphosphate, its active form, is similar to guanosine and is selectively toxic to infected cells as it is preferentially incorporated into viral DNA, reducing viral replication and controlling infections [206].

#### 5.5.3. Anti-HCMV Agent 1,2,4-Triazol-Quinoxalin Derivative

**Figure 21:**
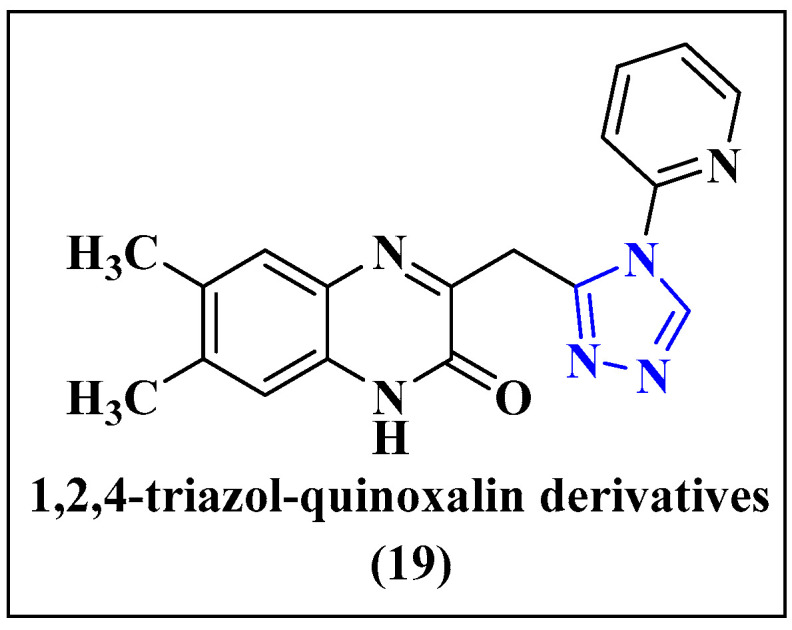
1,2,4-triazol-quinoxalin derivative (**19**), a new anti-human-cytomegalovirus (HCMV) agent.

Another potential anti-HCMV agent is represented by quinoxaline derivatives, which have been found in recent research studies. Quinoxaline is a heterocyclic compound containing a benzene ring fused to a pyrazine ring, and its derivatives have diverse biological activities, including antiviral properties. These compounds have exhibited greater antiviral activity against HCMV compared to the standard drug ganciclovir [208]. The triazole and quinoxaline moieties in 1,2,4-triazoloquinoxaline (Figure 21) have been reported to exhibit antiviral activity against HCMV. The triazole moiety is a five-membered heterocyclic ring containing three nitrogen atoms, which has been reported to possess antiviral activity. The quinoxaline moiety is a bicyclic aromatic ring system that has also been reported to exhibit antiviral activity. Studies have shown that 1,2,4-triazoloquinoxaline derivatives can inhibit HCMV replication by targeting the viral DNA polymerase, which is a key enzyme involved in viral replication. These compounds have also been reported to have low cytotoxicity toward human cells, making them potentially useful as antiviral agents [209].

**Scheme 13:**
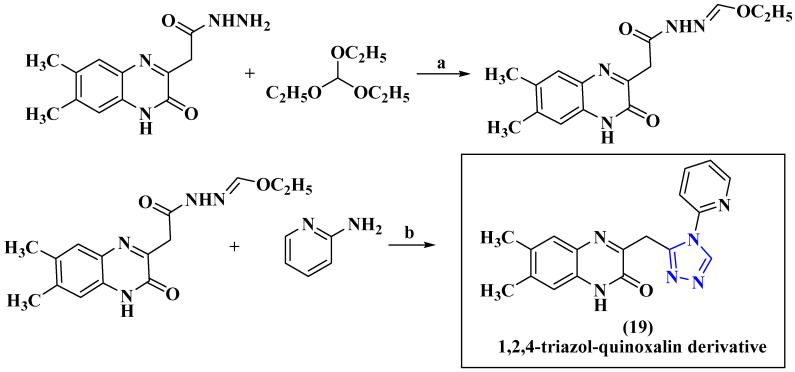
Synthesis of 1,2,4-triazol-quinoxalin derivative.

Key synthetic steps for Scheme 13 [210]: (a) The compound 2-(6,7-dimethyl-3-oxo-3,4-dihydroquinoxalin-2-yl)acetohydrazide was reacted with triethylorthoformate in ethanol to afford ethyl [(6,7-dimethyl-3-oxo-3,4- dihydroquinoxalin-2-yl)acetyl]hydrazonoformate; further, (b) treatment of the hydrazonoformate with 2-aminopyridine in acetic acid reflux afforded 6,7-dimethyl-3-{[4-(pyridin-2-yl)- 4H-1,2,4-triazol-3-yl]methyl}quinoxalin-2(1H)-one to obtain the product **19**.

Reagents and conditions: (a) C_2_H_5_OH, rt; (b) CH_3_COOH, reflux.

Mechanism of Action of 1,2,4-triazol-quinoxalin Derivatives: 1,2,4-triazol-quinoxalin derivatives have potential as antiviral agents against HCMV, but their exact mechanism of action is not fully understood. They may inhibit viral DNA replication and interfere with viral gene expression or virion assembly. Additionally, they may have immunomodulatory effects that enhance antiviral activity or inhibit immune evasion strategies against HCMV [211]. Valganciclovir is another available drug for the treatment of HCMV. The mechanism of action, ways of use and side effects of valganciclovir and ganciclovir are given in Table 16.

Some other synthesized compounds that show activity against HCMV are given in the Table 17.

### 5.6. Anti-HSV Agent

Lycogarubins have been reported as the first naturally occurring dimethyl pyrrole-dicarboxylate attached to two indole moieties [218]. These compounds were isolated from the fruit bodies of the slime molds *Arcyria denudate*, and are closely related to Arcyriarubins and Arycyriaflavins. Three novel dimethyl pyrrole dicarboxylates named Lycogarubins A–C were isolated by Haahimoto et al. from the *Myxomycetes Lycogala epidendrum*, among which Lycogarubin C showed the effective potency against HSV-I [219]. Idoxuridine, trifluridine and brivudine are marketed anti-HSV drugs used as ointments for the treatment of eye infections due to HSV. They act by inhibiting DNA polymerase of HSV and interrupting viral DNA synthesis. The mechanisms of action, ways of use, and side effects of idoxuridine, trifluridine and brivudine are given in Table 18**.**

Mechanism of Action of Anti-HSV Drugs: Anti-HSV drugs target the herpes simplex virus (HSV) and work by inhibiting viral replication and/or reducing the severity and duration of HSV symptoms. There are three main classes of anti-HSV drugs:

Nucleoside analogues: These drugs mimic the structure of the nucleotides that the virus needs to replicate its DNA. When the virus interacts with the DNA part of the nucleoside, it disrupts the replication process, preventing the virus from making new copies of it. Examples of nucleoside derivatives used to treat HSV include acyclovir, valacyclovir, and famciclovir [58].

Non-nucleoside inhibitors: These drugs target specific viral enzymes that are essential for viral replication. They work by binding to the enzyme and blocking its activity, thereby preventing the virus from replicating. Examples of non-nucleoside inhibitors used to treat HSV include foscarnet and cidofovir [222].

Interferons: These drugs are proteins that the body naturally produces in response to viral infections. They provoke activity by stimulating the immune system to produce antiviral proteins that can inhibit viral replication. Examples of interferons used to treat HSV include interferon alpha and interferon beta [223]. Here, it is important to note that while these drugs can help reduce the severity and duration of HSV symptoms, they do not cure the infection. The virus remains in the body and can reactivate, causing recurrent outbreaks.

### 5.7. Anti-Ebola Agent

**Figure 22:**
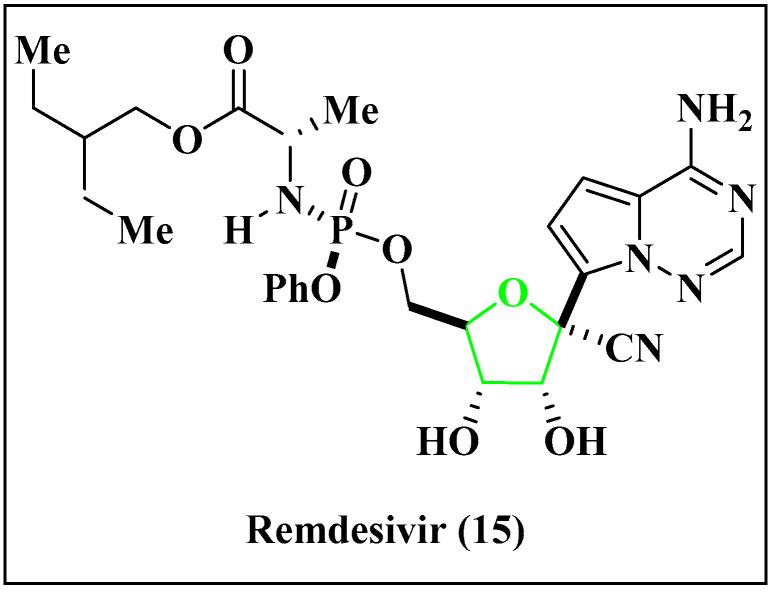
Anti Ebola agent remdesivir (**15**).

Anti-Ebola agents are drugs that target the Ebola virus by preventing its replication or entry into human cells. Examples include ZMapp, a combination of three monoclonal antibodies, and remdesivir (Figure 22) [224,225], which is used as a broad-spectrum antiviral drug. The other treatments developed include RNA-based therapies and gene therapies. Such advanced treatments offer genuine hope for a better future, in which EVD will not be considered to be a major concern of public health.

**Scheme 14:**
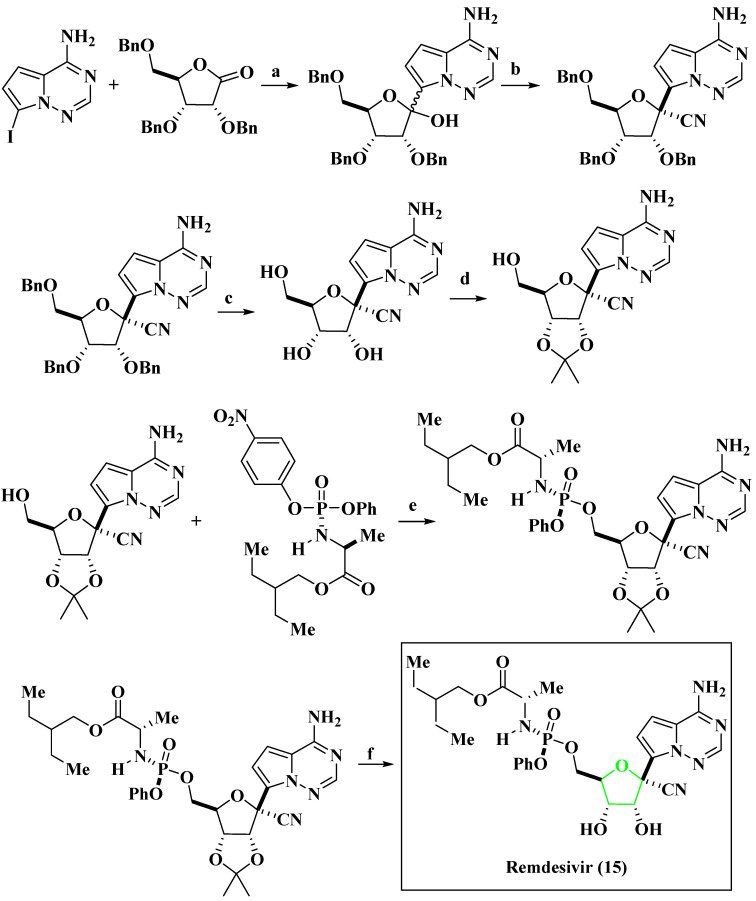
Synthesis of remdesivir.

Key synthetic steps for Scheme 14 [226]: (a) The iodopyrazole was dissolved in THF and cooled to 0 °C, TMSCl was added, and after 1 h, phenylmagnesium chloride was added. The reaction mixture was cooled to −20 °C and iso-propylmagnesium chloride was added slowly to (b) a pre-cooled (−40 °C.) solution of (3*R*,4*R*,5*R*)-2-(4-aminopyrrolo[2,1-f][1.2.4]triazin-7-yl)-3,4-bis(benzyloxy)-5-((benzyloxy)methyl)tetrahydrofuran-2-ol in DCM trifluoroacetic acid was added, followed by a pre-cooled (−30 °C.) solution of TMSOTf and TMSCN in DCM at rt; (c) the tribenzyl cyano nucleoside was dissolved in anhydrous CH_2_Cl_2_ and cooled to about −20 °C. A solution of BCl_3_, the reaction mixture, was stirred for 1 h at about −20 °C. MeOH was added dropwise (d) to a mixture of (2*R*,3*R*,4S.5*R*)-2-(4-aminopyrrolo[2,1-f][1.2.4] triazin-7-yl)-3,4-dihydroxy-5-(hydroxymethyl)tetrahydrofuran-2-carbonitrile, 2,2-dimethoxypropane and acetone at ambient temperature, to which sulfuric acid was added. The mixture was warmed to about 45 °C and (e) N,N-dimethylacetamide was added to a mixture of (2R,3R,4S.5R)-2-(4-aminopyrrolo[2,1-f][1.2.4]triazin-7-yl)-3,4-dihydroxy-5-(hydroxymethyl)tetrahydrofuran-2-carbonitrile, (S)-2-ethylbutyl2-(((*S*)-(4-nitrophenoxy)(phenoxy)phosphoryl)amino)propanoate and MgCl_2_. Then the resulting reaction mixture was warmed at 30 °C with constant stirring and N,N-diisopropylethylamine was added slowly, (f) the deprotection of the alcohols was performed by conc. HCl to obtain the product 15.

Reagents and conditions: (a) TMSCl, PhMgCl, *^i^*PrMgCl·LiCl, THF, −20 °C; (b) TMSCN, TMSOTf, TfOH, CH_2_Cl_2_, −78 °C; (c) (1) BCl_3_, CH_2_Cl_2_, −40 °C; (2) Et_3_N, MeOH, −78 °C–rt; (d) 2,2-DMP, H_2_SO_4_, Me_2_CO, rt; 45 °C; (e) MgCl_2_, DIPEA, MeCN, 50 °C; (f) 12 N HCl, THF (1:5), rt.

Mechanism of Action of Remdesivir: Remdesivir interferes with the Ebola virus’s replication by inhibiting its RNA-dependent RNA polymerase enzyme. It acts as a chain terminator, preventing the virus from replicating further and causing harm to the host [97]. The mechanism of action and ways of use are given in Table 19.

Synthesized compounds that show activity against the Ebola virus are given in Table 20.

### 5.8. Anti-SARS-COV-2 Agent

SARS-CoV-2 is a beta-coronavirus in the B lineage that is closely related to the SARS-CoV virus [234]. The major structural genes include N, S, SM and M, while an additional glycoprotein HE occurs in HCoV-OC43 and HKU1 beta-coronaviruses. SARS-CoV-2 shares 96% of its genome with a bat coronavirus. There are several types of anti-SARS-CoV-2 medications, each with its own mechanism of action. The examples are mentioned below:

Vaccines: Vaccines stimulate the immune system to produce antibodies that can neutralize the virus before it can cause an infection. There are currently several COVID-19 vaccines available, including mRNA vaccines, viral vector vaccines, and inactivated or protein subunit vaccines [235].

As of 12 January 2022, the following vaccines have been granted Emergency Use Listing:

- Comirnaty vaccine by Pfizer/BioNTech, approved 31 December 2020.
- SII/COVISHIELD and AstraZeneca/AZD1222 vaccines, approved 16 February 2021.
- Janssen/Ad26.COV 2.S vaccine developed by Johnson & Johnson, approved 12 March 2021.
- Moderna COVID-19 vaccine (mRNA 1273), approved 30 April 2021.
- Sinopharm COVID-19 vaccine, approved 7 May 2021.
- Sinovac-CoronaVac vaccine, approved 1 June 2021.
- Bharat Biotech BBV152 COVAXIN vaccine, approved 3 November 2021.
- Covovax (NVX-CoV2373) vaccine, approved 17 December 2021.
- Nuvaxovid (NVX-CoV2373) vaccine, approved 20 December 2021.

Monoclonal antibodies: Monoclonal antibodies are laboratory-made proteins that mimic the immune system’s ability to fight off harmful pathogens. They can neutralize the virus by binding to specific proteins on its surface and preventing it from entering host cells [236].

Antiviral drugs (Table 21): Antiviral drugs can inhibit viral replication by targeting specific viral proteins or enzymes. For example, remdesivir is an antiviral drug that inhibits the viral RNA polymerase enzyme which is essential for the replication of the virus [237].

Immune-modulators: Immune modulators help modulate the immune response to the virus. For example, dexamethasone is a corticosteroid drug that reduces inflammation and has been shown to improve outcomes in severe COVID-19 cases [238].

**Table 21:** Anti-SARS-CoV-2 drugs.

Sl. No.	Drug Name	Drug Target	Mechanism of Action	Ways of Use	Side Effect	Brand Name
1.	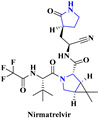	SARS-CoV-2 [239]	Nirmatrelvir inhibits cysteine residue in the 3C-like protease (3CL^PRO^) of SARS-CoV-2	Oral	There is no such side effect observed	Paxlovid
2.	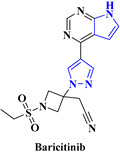	COVID-19 [240]	Baricitinib inhibits the activity of JAK proteins and modulates the signaling pathway of various interleukins, interferons	Oral	There is no such side effect observed	Olumiant

Some other synthesized compounds that show activity against SARS-COV-2 are given in Table 22.

### 5.9. Anti-HPV Agent

HPV can infect both men and women, and it is predicted that most sexually active adults will become infected at some point in their lives. It can cause genital warts and certain types of cancer, including cervical, anal and oropharyngeal cancer [247]. There is no cure for HPV; however, there are various treatment options available (as below) to minimize the symptoms in a controlled way [248].

Imiquimod (Aldara): This topical cream stimulates the immune system to fight the virus and is used to treat external genital warts and certain pre-cancerous skin lesions caused by HPV.

Podofilox (Condylox): This topical solution works by destroying the skin cells infected with HPV and is used to treat external genital warts.

Trichloroacetic acid (TCA): This topical solution is used to treat genital warts and certain pre-cancerous skin lesions caused by HPV.

Cidofovir (Vistide): This antiviral drug is used to treat severe cases of HPV infections, including those that have spread to other parts of the body.

Gardasil and Cervarix: These are vaccines that protect against several strains of HPV, including those that are known to cause most cases of cervical cancer.

A synthesized compound that shows activity as an anti-HPV agent is given in Table 23**.**

### 5.10. Anti-Rabies Agent

There are two main ways to prevent and treat rabies virus infection: vaccination and post-exposure prophylaxis (PEP) with immunoglobulin and vaccines. In the case of suspected rabies virus exposure, PEP is recommended to prevent the virus from causing an infection. PEP typically involves the administration of both rabies immunoglobulin (RIG), which contains antibodies against the virus, and a series of rabies vaccine injections [250].

### 5.11. Anti-Zika Agent

There is currently no specific antiviral treatment for Zika virus infection, and treatment is generally supportive. For example, drugs that are used to treat other Flaviviruses, such as dengue and yellow fever, are being tested in clinical trials to see if they can also be effective against the Zika virus [251,252]. In addition, multiple vaccine candidates, such as DNA vaccines and RNA vaccines, are in various stages of development. These vaccines are being tested in preclinical and clinical trials to determine their safety and effectiveness in preventing Zika virus infection [253].

Synthesized compounds that show activity against the Zika virus are given in Table 24.

Several compounds have shown a broad inhibition activity against Flavivirus proteases and have been extensively studied.

### 5.12. Anti-Polio Agent

Remediation of polio involves immunization through the administration of the oral polio vaccine (OPV) or the inactivated polio vaccine (IPV). OPV is the preferred vaccine for most countries, as it is easy to administer and can also provide herd immunity by interrupting the transmission of the virus from person to person [261].

### 5.13. Anti-West Nile Agent

There is currently no specific treatment or vaccine for West Nile virus, but several vaccines are being developed and tested in clinical trials. Nonetheless, several promising vaccine candidates are currently being studied, and ongoing research in this area is very promising and provides hope for the future [262].

### 5.14. Anti-Chickenpox Agent

Remediation for chickenpox includes management of the symptoms, such as the use of antihistamines to alleviate itching and pain relievers to reduce fever. There are two major ways to prevent and treat chickenpox: through vaccination and antiviral medications. The chickenpox vaccine is a live, attenuated vaccine that contains a weakened form of the varicella–zoster virus [263]. Antiviral medications such as acyclovir, valacyclovir, and famciclovir can also be used to treat chickenpox. These drugs work by inhibiting the replication of the virus and are typically used in individuals who are at high risk of complications, such as pregnant women, immunocompromised individuals, and those with severe symptoms [264].

### 5.15. Anti-Influenza Agent

The most commonly used drugs for the treatment of the flu disease are neuraminidase inhibitors, which generally work by blocking the intracellular spread of the virus in the body. The two main neuraminidase inhibitors used for this purpose are oseltamivir (Tamiflu) [265] and zanamivir (Relenza) [266]. These drugs are effective in reducing the duration and severity of flu symptoms, as well as preventing complications. 

Synthesized compounds with prominent activity against the influenza virus are mentioned in Table 25.

### 5.16. Anti-Yellow Fever Agent

Currently, there is no specific antiviral drug available to treat yellow fever. Treatment is primarily supportive, with measures such as fluid replacement, pain relief and management of other symptoms [272].

## 6. Conclusions

In this review article, we have precisely discussed the antiviral activities of structurally diverse oxa- and aza-cycles with respect to different diseases. We have highlighted the role of representative small molecules, from natural products to synthetic compounds with heterocyclic subunits, and demonstrated their antiviral features. Taking into consideration of the severity of the viral infections, it is undoubtedly necessary to have a complete data set, along with the structure–activity relationship (SAR) of various drug candidates against the infectious viruses. In this regard, this review article could definitely play a crucial role in the discovery of antiviral drugs.

## 7. Scope, Limitation and the Presentation of the Future Trend of Antiviral Drugs

Antiviral drugs have revolutionized the treatment and control of infections caused by viral diseases. They target specific viral mechanisms, by such means as inhibiting replication, preventing viral entry into cells and blocking the activity of the viral enzyme. These drugs have significantly improved patient outcomes and reduced the spread of contagions. They are capable enough to tackle infections like HIV, hepatitis, influenza, herpes, and more. In spite of the suitability of such drugs in terms of the proper treatment and control of viral infections, they do have limitations. Viruses can develop resistance to certain drugs, necessitating the development of new classes of drugs or combination therapies. Moreover, antiviral drugs may cause side effects and interact with other medications, a situation which requires careful management. The challenges are even greater when facing the newly emerged viruses, and the lack of specific and effective treatment options becomes apparent. The development of drugs targeting such viruses is very complicated and involves extensive research and development, advanced computational support and dedicated clinical trials. Additionally, viral mutations and the potential for drug resistance further impede the effectiveness of existing therapies against newly arrived viruses.

Furthermore, emerging technologies like CRISPR/Cas9-based gene editing hold the potential for targeted viral genome disruption, offering innovative approaches to combat viral infections. The ongoing research and development of antiviral drugs aim to address current limitations, including drug resistance, side effects and access issues, while providing more effective, safer and accessible treatments for viral infections in the future.

## Figures and Tables

**Table 1:** SAR analysis of darunavir.

Compound	Substitution		Activity	
	−R^1^	−R^2^	Inhibition Concentration (nM)	Inhibitor Constant (K_i_)
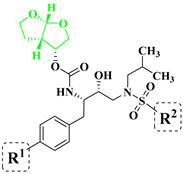	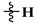	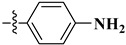	IC_90_ = 4.1	16 pM
	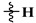	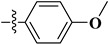	IC_90_ = 1.4	14 pM
	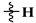	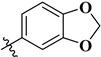	IC_50_ = 0.22	14 pM
	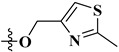	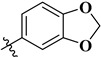	IC_50_ = 0.7	15 fM
	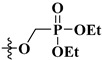	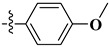	IC_50_ = 1	8 pM

**Table 2:** SAR analysis of amprenavir.

Compound	Substitution		Activity	
	−R^1^	−R^2^	EC_50_ (nM)	Inhibitor Constant (K_i_) (nM)
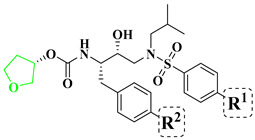	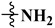	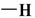	2.93	0.84
	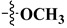	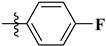	4.29	5.8
	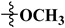	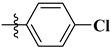	2.32	6.4
	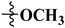	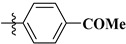	6.89	5.8
	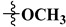	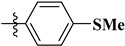	6.04	7.4
	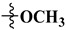	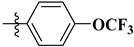	3.63	5.2
	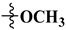	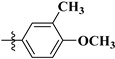	5.53	1.8
	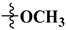	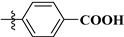	4.30	1.9
	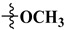	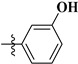	3.46	4.3
	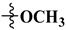	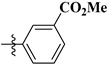	9.2	1.6
	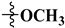	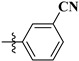	4.48	8.1
	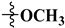	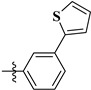	3.08	3.5
	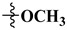	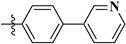	2.68	1.6
	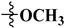	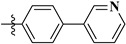	2.25	3.8
	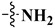	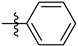	0.10	1.95
	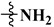	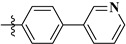	0.10	0.33
	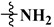	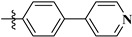	2.93	0.086

**Table 3:** Anti-HIV drugs.

Sl. No.	Drug Name	Drug Target	Mechanism of Action	Ways of Use	Side Effect	Brand Name
1.	Zalcitabine [135]	Reverse transcriptase/RNase H (Human immunodeficiency virus 1)	Inhibiting reverse transcriptase and terminating the viral DNA chain	Oral	Liver failure, inflammation of the pancreas	Hivid
2.	Amprenavir [136]	Human immunodeficiency virus type 1 protease	Blocks the active site of HIV protease to prevent cleavage of the viral precursor proteins	Oral	Diarrhea, kidney failure, weakness	Agenerase
3.	Maraviroc [137]	C-C chemokine receptor type 5	Blocks GP120-CCR5 interaction to inhibit HIV entry	Oral	Liver problems, nausea, allergic reactions	Selzentry
4.	Tenofovir disoproxil Fumarate [138]	Reverse transcriptase/RNaseH (Human immunodeficiency virus 1)	Competes with dATP and inhibits the activity of HIV RT	Oral	Trouble sleeping, dizziness, diarrhea	Viread
5.	Emtricitabine [139]	Reverse transcriptase/RNase H (Human immunodeficiency virus 1)	Through nucleoside reverse transcriptase inhibition	Oral	Body aches, ough, diarrhea, fever	Truvada
6.	Cabotegravir [140]	Integrase (Human immunodeficiency virus 1)	By HIV-1 integrase inhibition	Oral	Abnormal dreams, dark urine, difficulty in breathing	Vocabria
7.	Lenacapavir [141]	Gag-Pol polyprotein	Lenacapavir works by blocking the HIV-1 virus’ protein shell (the capsid), thereby interfering with multiple essential steps of the viral lifecycle	Oral/subcutaneous injection	Nausea	Sunlenca

**Table 4:** Synthesized anti-HIV compounds.

Sl. No.	Antiviral Agent	Drug Target	Activity
1.	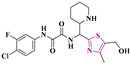	HIV [142]	Methylthiazole derivatives effectively inhibit HIV-1 entry into cells, demonstrating low micromolar potency (IC_50_ = 1.6 µM) and a favorable selectivity index (SI) in both single-cycle TZM-bl cell assays and multicycle MT-2 cell assays.
2.	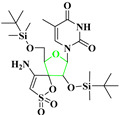	HIV-1 and HIV-2 [143]	3″-alkenyl-substituted TSAO derivatives exhibit anti-HIV-1 and anti-HIV-2 activity at subtoxic concentrations against HIV-2 RT involved the RNA-dependent DNA polymerase assay with EC_50_(HIV-1_MT-4_) = 0.06 ± 0.03 µM and EC_50_(HIV-2_MT-4_) >10 µM.
3.	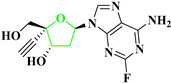	HIV [144]	4′-ethynyl-2-fluoro-2′-deoxyadenosine demonstrates exceptional potency in inhibiting HIV-1 replication in phytohemagglutinin-activated peripheral blood mononuclear cells with EC_50_(HIV-1_NL4-3_) = 50 pM.
4.	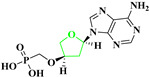	HIV-1 [145]	Deoxythreosyl phosphonate nucleosides exhibit potent anti-HIV-1 activity (EC_50_(HIV-1) = 2.53 μM) by binding effectively to the active site pocket of HIV-1 reverse transcriptase. These compounds show no cytotoxicity, even at high concentrations (CC_50_ > 316 µM). The incorporation kinetics of these compounds into DNA were studied using their diphosphate form as a substrate and HIV-1 reverse transcriptase as the catalyst.
5.	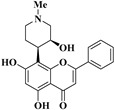	HIV-1 [146]	Flavopiridol, a cyclin-dependent kinase (CDK) inhibitor, blocks HIV-1 Tat transactivation and viral replication by inhibiting P-TEFb kinase activity. The cytotoxicity of Flavopiridol and its analogues was evaluated using an MTT-based cell viability assay, demonstrating their potential as anti-HIV-1 therapeutics with EC_50_ = 7.4 nM.

**Table 5:** SAR analysis of asunaprevir derivatives.

Compound	Substitution				Activity
	−R^1^	−R^2^	−R^3^	−R^4^	IC_50_ (nM)
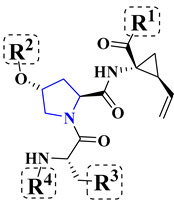	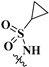	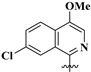	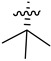	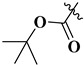	2
	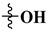	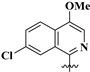	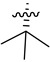	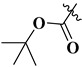	247
	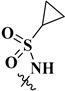	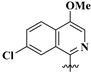	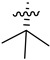	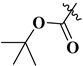	2
	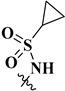	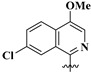	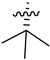	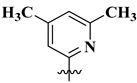	4
	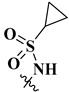	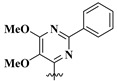	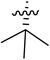	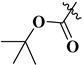	4
	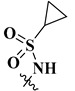	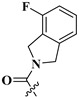	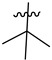	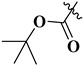	2
	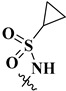	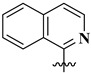	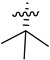	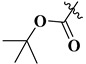	7
	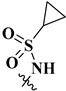	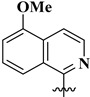	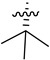	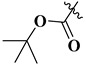	1
	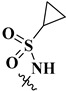	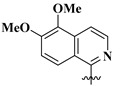	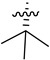	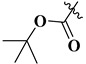	7
	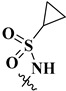	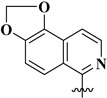	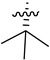	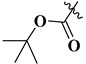	2

**Table 6:** SAR analysis of 3-(1,2,4-oxadiazole)-quinolone derivatives.

Compound	Ubstitution		Activity
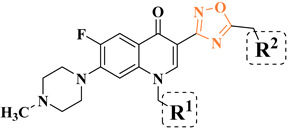	**−R^1^**	**−R^2^**	**IC_50_ (μM)**
	4-Cl-C_6_H_4_	4-F_4_-C_6_H_4_	0.019
	4-Cl-C_6_H_4_	2,4-DiF-C_6_H_3_	0.075
	4-CF_3_-C_6_H_4_	C_6_H_5_	0.024
	2-F,4-CF_3_-C_6_H_3_	C_6_H_5_	0.014
	2-F,4-CF_3_-C_6_H_3_	4-F_4_-C_6_H_4_	0.015

**Table 7:** SAR Analysis of grazoprevir derivatives.

Compound	Substitution			Activity	
	−R^1^	−R^2^	−R^3^	EC_50_ (nM)	Inhibitor Constant (K_i_) (nM)
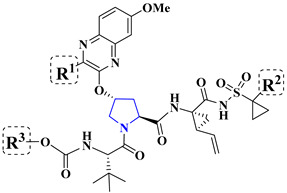	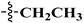	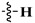	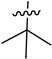	24	19 ± 2.7
	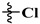	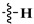	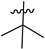	6.6	7.8 ± 1.1
	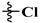	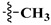	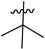	6.3	6.1 ± 1.1
	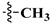	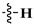	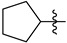	10	9.2 ± 0.9
	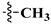	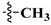	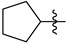	4.5	7.1 ± 1.1
	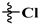	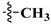	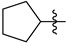	3.1	3.9 ± 0.7

**Table 8:** Anti-HCV drugs.

Sl. No.	Drug Name	Drug Target	Mechanism of Action	Ways of Use	Side Effect	Brand Name
1.	Asunaprevir [147]	Genome polyprotein (Hepatitis C virus genotype 1b)	Inhibits the proteolytic activity of HCV NS3/4A protease	Oral	Flu-like symptoms, skin rash, irritability, headache	Sunvepra
2.	Boceprevir [163]	NS3/4A protein (Hepatitis C Virus)	Inhibits the proteolytic activity of HCV NS3/4A protease	Oral	Vomiting, dry skin, fever, sore throat	Victrelis
3.	Grazoprevir [164]	NS3/4A protein (Hepatitis C Virus)	Inhibits the proteolytic activity of HCV NS3/4A protease	Oral	Headache, nausea, trouble breathing	Zepatier
4.	Sofosbuvir [165]	RNA-dependent RNA-polymerase (Hepatitis C Virus)	Inhibits RNA-dependent RNA polymerases of HCV NS5B (non-structural protein 5B)	Oral	Fatigue, headache, decreased appetite	Vosevi
5.	Daclatasvir [166]	Nonstructural protein 5A (Hepatitis C Virus)	Disrupts the NS5A proteins that have undergone hyperphosphorylation; interferes with the functioning of newly formed HCV replication complexes.	Oral	Headache, feeling tired, nausea	Daklinza

**Table 9:** Synthesized anti-HCV compounds.

Sl. No.	Antiviral Agent	Drug Target	Activity
1.	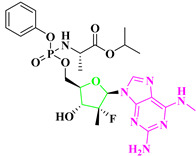	Hepatitis C Virus (HCV) [167]	The fatty acid synthase inhibitors showed good antiviral activity in a cell-based HCV replicon assay and an acceptable selectivity index. Their observed cell permeability in an MDCK permeability assay supports these findings. The antiviral activities align with the biochemical inhibition (IC_50_ values > 30 μM) of Hfasn [168].
2.	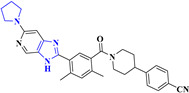	HCV [169]	Imidazo[2,1-b]thiazole targets HCV NS4B, specifically the second amphipathic α helix (4BAH2). The effectiveness of the compound against Huh-7 cells was assessed, screening an EC_50_ value of 18 nM [170].
3.	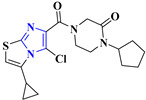	HCV [171]	The efficacy of the compound against Huh-7 cells shows promising activity, which reflects in the EC_50_ value (16 nM).

**Table 10:** SAR analysis of entecavir derivatives.

Compound	Substitution		Activity
	−R^1^	−R^2^	IC_50_ (μM)
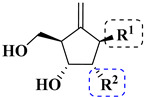	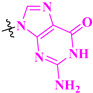	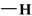	0.03
	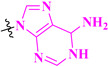	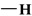	0.128
	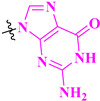	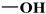	>100

**Table 11:** SAR analysis of dehydro-andrographolide and andrographolide derivatives.

Compound	Substitution	Activity
	−R	IC_50_ (μM)
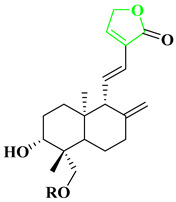	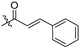	18
	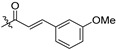	1970
	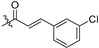	207
	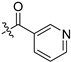	40
	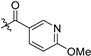	2313
	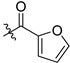	19.2
	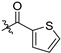	162
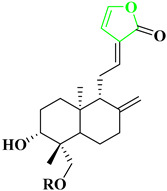	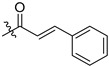	210
	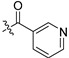	1137
	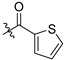	121
	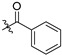	880
	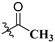	280
	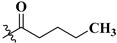	565
	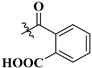	1317

**Table 12:** Anti-HBV drugs.

Sl. No.	Drug Name	Drug Target	Mechanism of Action	Ways of Use	Side Effect	Brand Name
1.	Entecavir [185]	DNA polymerase	Inhibits the activity of HBV DNA polymerase	Oral	Headache, dizziness, nausea	Baraclude
2.	Telbivudine [186]	DNA polymerase	Inhibits the activity of HBV DNA polymerase	Oral	Diarrhea, cough, headache, dizziness	Tyzeka
3.	Lamivudine [187]	Protein P (HBV-F)	Inhibits reverse transcriptase	Oral	Nausea, diarrhea, headaches	Lamivudine

**Table 14:** Anti-RSV drugs.

Sl. No.	Drug Name	Drug Target	Mechanism of Action	Ways of Use	Side Effect	Brand Name
1.	Ribavirin [198]	RNA-directed RNA polymerase L (HPIV-2)	Targets viral RNA polymerase to inhibit mRNA synthesis	Oral or inhaled	Hemolytic anemia, asthenia, rigors, fevers	Rebetol
2.	RSV-IGIV [199]	RSV G protein	Prevents the binding of RSV surface glycoproteins F and G	-	-	RespiGam
3.	Palivizumab [200]	Fusion glycoprotein F0 (Human respiratory syncytial virus B)	Prevents the binding of RSV surface glycoprotein F	Injection	Sore throat, runny nose, vomiting	Synagis

**Table 15:** Synthesized anti-RSV compounds.

Sl. No.	Antiviral Agent	Drug Target	Activity
1.	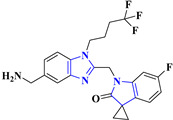	Respiratory syncytial virus [201]	The anti-RSV property of the compound tested with Hep2 cells with IC_50_ = 1.2 nM. The cell viability was measured using MTT reagents and cell cytotoxicity was evaluated through parallel assessments with plaque reduction assays.
2.	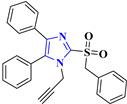	Respiratory syncytial virus [202]	The screening of the compounds against nonattenuated respiratory syncytial virus was done by high-throughput protocol with EC_50_ = 0.36–0.55 μM.

**Table 16:** Anti-HCMV drugs.

Sl. No.	Drug Name	Drug Target	Mechanism of Action	Ways of Use	Side Effects	Brand Name
1.	Valganciclovir [212]	DNA polymerase	Inhibits the activity of viral DNA polymerase	Oral	Diarrhea, upset stomach, dizziness	Valcyte
2.	Ganciclovir [213]	DNA polymerase catalytic subunit	DNA polymerase to inhibit viral DNA synthesis	Oral	Diarrhea, loss of appetite, increased sweating	Cytovene

**Table 17:** Synthesized anti-HCMV compounds.

Sl. No.	Antiviral Agent	Drug Target	Activity
1.	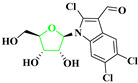	HCMV [214]	Synthesized trichlorinated indole nucleosides were tested for activity against human cytomegalovirus (HCMV). Cytotoxicity was assessed using two methods: microscopic inspection of uninfected HFF cells [215] and crystal violet staining with spectrophotometric quantitation in KB cells [216]. The IC_50_ was found to be 0.23 µM.
2.	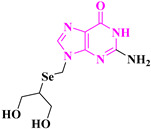	HCMV [217]	The compound was assayed for antiviral activity against HCMV (Davis, VR-807) cells in a cytotoxic assay. The EC_50_ value of 53.1 µM showed only moderate antiviral activity against HCMV.

**Table 18:** Anti-HSV drugs.

Sl. No.	Drug Name	Drug Target	Mechanism of Action	Ways of Use	Side Effect	Brand Name
1.	Idoxuridine [95]	DNA polymerase	HSV DNA polymerase to inhibit viral DNA synthesis	Used as an ointment	Eye irritation or pain, swelling of the eye	Dendrid
2.	Trifluridine [220]	DNA polymerase	Inhibits HSV DNA replication	Eye drop	Eye pain, mild burning of eyes	Viroptic
3.	Brivudine [221]	DNA polymerase	Inhibits HSV DNA replication	Oral	No side effect	Zostex

**Table 19:** Anti-EBOV drugs.

Sl. No.	Drug Name	Drug Target	Mechanism of Action	Ways of Use	Side Effect	Brand Name
1.	BCX4430 [227]	RNA-directed RNA polymerase L	Acts as a nonobligate RNA chain terminator upon incorporation into viral RNA	Intramuscular or oral	-	BioCryst
2.	Neplanocin A [228]	S-adenosyl-l-homocysteine (SAH) hydrolase inhibitor	-	-	-	-
3.	Lectins [229]	-	-	-	-	-
4.	Remdesivir [230]	RNA-directed RNA polymerase L	Inhibits the viral RNA polymerase enzyme, which is essential for the replication of the virus	Intravenously	Nausea, vomiting, diarrhea, and elevated liver enzymes	Veklury
5.	TKM-130803 [231]	mRNA of EBOV	Degrades the viral RNA of the Ebola virus	Intravenous infusion	Immune reactions, off-target effects, and toxicity due to high doses	-

**Table 20:** Synthesized anti-Ebola compounds.

Sl. No.	Antiviral Agent	Drug Target	Activity
1.	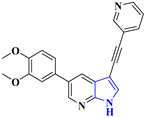	Ebola virus [232]	3,5-Disubstitutedpyrrolo[2,3 b]pyridines demonstrate potent activity against DENV-infected primary dendritic cells and exhibit anti-EBOV activity. A LanthaScreen binding assay was conducted to assess their effectiveness against the Ebola virus (EC_50_ = 0.59 μM).
2.	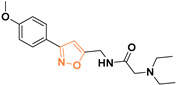	Ebola virus [233]	Isoxazole analogues were tested as inhibitors of Ebola GP-mediated cell entry, with a promising IC_50_ value in the range of 30 μM.
3.	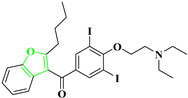	Ebola virus [200]	-

**Table 22:** Synthesized anti-SARS-COV-2 compounds.

Sl. No.	Antiviral Agent	Drug Target	Activity
1.	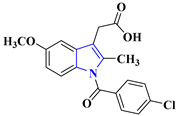	SARS-CoV-2 [241]	Indomethacin exhibits potent antiviral activity against SARS coronavirus by selectively inhibiting viral RNA synthesis, independent of its COX inhibition and anti-inflammatory properties [242], with a promising EC_50_ value of 50 μM.
2.	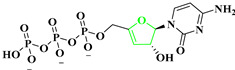	SARS-CoV-2 [243]	3′-Deoxy-3′,4′-didehydro-cytidine triphosphate (ddhCTP) is a novel antiviral molecule that specifically targets non-native RNA polymerases. Its production does not affect cell viability or growth rate.
3.	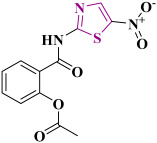	SARS-CoV-2 [244]	Nitazoxanide (NTZ), originally an antiparasitic agent, shows potent activity against various RNA and DNA viruses, including SARS-CoV-2 (EC_50_ = 3.16 μM). Further studies are needed to understand its mode of action and specificity [245].
4.	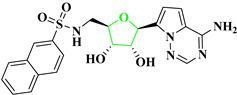	SARS-CoV-2 [246]	This compound targets the SARS-CoV-2 methyltransferases MTase and Nsp14. Which demonstrates superior anti-SARS-CoV-2 inhibition (EC_50_ = 0.72 μM), as evidenced by the compound’s high antiviral activity and low cytotoxicity.

**Table 23:** Synthesized anti-HPV compound.

Sl. No.	Antiviral Agent	Drug Target	Activity
1.	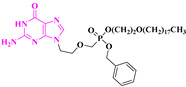	Human papillomavirus (HPV-18) [249]	The compound octadecyloxyethyl benzyl 9-[(2-phosphonomethoxy)ethyl]guanine effectively inhibited the amplification of HPV-11 plasmid DNA in transfected cells (EC_50_ = 0.10 μM). Cell viability was assessed using CellTiter-Glo reagent (Promega) and measured with a luminometer.

**Table 24:** Synthesized anti-Zika compounds.

Sl. No.	Antiviral Agent	Drug Target	Activity
1.	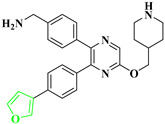	Zika virus [254]	These compounds have demonstrated significant efficacy in both cellular and in vivo studies, particularly in U87 glioma cells infected with the ZIKV FLR strain [255], with IC_50_ values ranging from 200–790 nM. Their mode of action involves binding to an allosteric pocket of NS3, which is a viable target within the Flavivirus protease. This binding occurs in contrast to the shallow active site, which typically recognizes polar and positively charged residues in the substrate, such as arginine (Arg) or lysine (Lys) [256].
2.	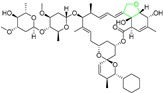	Zika virus [257]	Doramectin has been investigated as a potential broad-spectrum antiviral agent against ZIKV. It has shown strong inhibitory effects against ZIKV infection in SNB19 cells. It shows an EC_50_ value of less than 3 μM.
3.	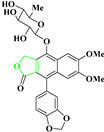	Zika virus [258]	Another substituted Glycosylated diphyllin has shown strong inhibitory effects against ZIKV infection in various cell lines, including CHME3 cells (human microglia cells), with IC_50_ values ranging from 10–70 Nm.
4.	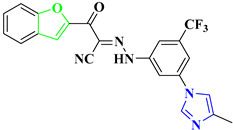	Zika virus [259]	Cyanohydrazones have also exhibited noteworthy antiviral activity against ZIKV, with an IC_90_ value of 4.2 ± 0.2. These compounds target the E-mediated membrane fusion process, effectively inhibiting ZIKV infection [260].

**Table 25:** Synthesized anti-influenza compounds.

Sl. No.	Antiviral Agent	Drug Target	Activity
1.	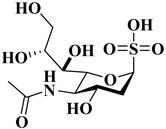	Influenza virus [267]	Sialosyl sulfonate is a potent inhibitor of influenza virus replication. Sialosyl sulfonates have shown their ability to block influenza A virus H3N2 (Perth/16/2009) infection of MDCK cells in vitro, using an in-situ ELISA method with IC_50_ > 1000 μM.
2.	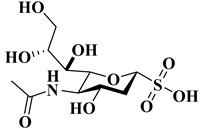	[267]	The above-mentioned method (Table 25, Sl. No. 1) was used to determine the activity of the compound. The IC_50_ value was found to be 0.7 μM.
3.	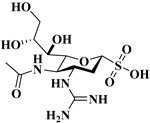	[267]	The above-mentioned method (Table 25, Sl. No. 1) was used to determine the activity of the compound. It showed an IC_50_ > 0.02 μM.
4.	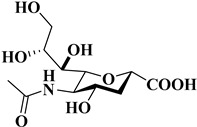	[267]	The above-mentioned method (Table 25, Sl. No. 1) was used to determine the activity of the compound. The IC_50_ value for this compound was found to be 550 μM.
5.	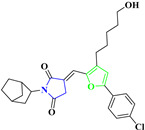	Influenza virus [268]	Through intervention in these host–virus interactions, the substituted furan-succinimide derivative can successfully impede viral replication and restrict the advancement of the infection, with the EC_50_ value determined to be 50 pM [269].
6.	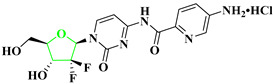	Influenza virus [270]	In human cells (HeLa), the compound was evaluated for its antiviral activity (EC_90_ = 11.4–15.9 μM), more precisely the polymerase activity of influenza A virus PR8. To assess this, a negative-sense EGFP gene, flanked with 5′ and 3′ UTRs derived from the NS segment, was cloned under the control of the human RNA polymerase I promoter.
7.	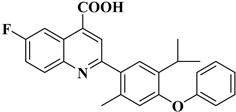	Influenza virus [271]	The compound inhibits human dihydroorotate dehydrogenase (DHODH) and viral replication of WSN-Influenza, with an EC_50_ of 41 nM.

## Abbreviations

**Abbreviation**	**Full Name**
DNA	Deoxyribonucleic Acid
RNA	Ribonucleic Acid
AIDS	Acquired Immune Deficiency Syndrome
HIV	Human Immunodeficiency Virus
HCV	Hepatitis C Virus
HBV	Hepatitis B Virus
RSV	Respiratory Syncytial Virus
HCMV	Human Cytomegalovirus
HSV	Herpes Simplex Virus
EBOV	Ebola Virus
SARS-CoV-2	Severe Acute Respiratory Syndrome CoV-2
PNBA	Para Nitro Benzoic Acid
GP120-CCR5	Beta chemokine receptors
dATP	Deoxyadenosine triphosphate
mRNA	Messenger Ribonucleic Acid
eIF4A	Eukaryotic initiation factor 4A
ToS	Toluenesulfonyl
OiPr	Isopropoxide
OSBT	O-(Tert-Butyldimethylsilyl)hydroxylamine
Boc	tert-butoxycarbonyl
PMP	Polymethylpentene
OBn	Benzyl group
mCPBA	meta-chloroperoxybenzoic acid
DMSO	Dimethyldioxirane
TFAA	Trifluoroacetic anhydride
TFA	Trifluoroacetic acid
DSC	N,N′-Disuccinimidyl carbonate
DEC	Diethylcarbamazine
DMAP	4-Dimethylaminopyridine
LDA	Lithium diisopropylamide
DDQ	2,3-Dichloro-5,6-Dicyanobenzoquinone
THF	Tetrahydrofuran
NBS	N-Bromosuccinimide
DPPA	Diphenylphosphoryl azide
HATU	Hexafluorophosphate Azabenzotriazole Tetramethyl Uronium
DCM	Dichloromethane
DMAc	N,N-Dimethylacetamide
DBU	1,8-Diazabicyclo(5.4.0)undec-7-ene
CDI	Carbonyldiimidazole
CPME	Cyclopentyl methyl ether
DIAD	Diisopropyl azodicarboxylate
IPAc	Isopropyl acetate
DMF	Dimethylformamide
DCC	N,N′-Dicyclohexylcarbodiimide
DEAD	Diethyl azodicarboxylate
TBAF	Tetra-n-butylammonium fluoride
4Å MS	4Å Molecular Sieve
